# Stimuli‐Responsive Catenane‐Based Catalysts

**DOI:** 10.1002/anie.202216787

**Published:** 2022-12-28

**Authors:** Adrien Bessaguet, Quentin Blancart‐Remaury, Pauline Poinot, Isabelle Opalinski, Sébastien Papot

**Affiliations:** ^1^ University of Poitiers UMR CNRS 7285 Institut de Chimie des Milieux et Matériaux de Poitiers (IC2MP) 4 rue Michel-Brunet, TSA 51106 86073 Poitiers cedex 9 France

**Keywords:** Catenane, Click Chemistry, Off/on Catalysts, Self-Immolative Linker, Signal Amplification

## Abstract

Rotaxanes and molecular knots exhibit particular properties resulting from the presence of a mechanical bond within their structure that maintains the molecular components interlocked in a permanent manner. On the other hand, the disassembly of the interlocked architecture through the breakdown of the mechanical bond can activate properties which are masked in the parent compound. Herein, we present the development of stimuli‐responsive Cu^I^‐complexed [2]catenanes as OFF/ON catalysts for the copper‐catalyzed alkyne‐azide cycloaddition (CuAAC) reaction. The encapsulation of the Cu^I^ ion inside the [2]catenanes inhibits its ability to catalyze the formation of triazoles. In contrast, the controlled opening of the two macrocycles induces the breaking of the mechanical bond, thereby restoring the catalytic activity of the Cu^I^ ion for the CuAAC reaction. Such OFF/ON catalysts can be involved in signal amplification processes with various potential applications.

## Graphical abstract

Cu^I^‐complexed [2]catenane‐based catalysts that can be activated ‘on‐demand’ through the controlled breakdown of the mechanical bond have been developed. Encapsulation of the Cu^I^ ion within the cavity formed by the two interlocked macrocycles inhibits its ability to catalyze the CuAAC reaction. However, the disassembly of the interlocked architecture by the opening of the macrocycles restores the capacity of the copper catalyst to form triazoles.

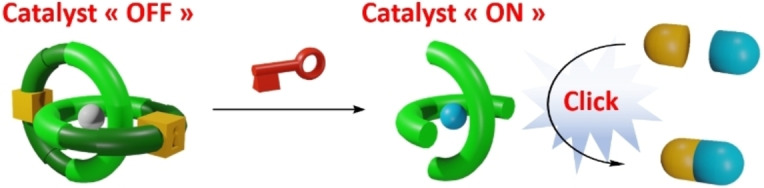

Mechanically interlocked molecules (MIMs) such as rotaxanes, catenanes and molecular knots exhibit unique properties that arise from the presence of a mechanical bond^[1]^ within their structure. Aside the development of efficient methods for the synthesis of MIMs,^[2]^ some attention has been paid to the controlled disassembly of the interlocked molecular architecture, principally for biomedical applications.^[3]^ “Nanovalves” grafted onto mesoporous silica nanoparticles,^[4]^ biodegradable polyrotaxanes,^[5]^ light‐^[6]^ and enzyme‐responsive^[7]^ rotaxane‐based prodrugs have been studied as potential drug delivery systems. Either chemically or enzymatically cleavable rotaxanes have also been investigated as probes for the detection and diagnosis of some diseases.^[8]^ Herein, we report on a novel approach that takes advantage of the controlled breakdown of a mechanical bond for the development of OFF/ON catalysts (Figure 1). Within this framework, we designed Cu^I^‐complexed [2]catenanes[^9^, ^10^] **1** including stimuli‐sensitive triggers and phenanthroline‐derived self‐opening macrocycles.^[7c]^ The encapsulation of Cu^I^ inside the cavity formed by the two interlocked macrocycles results in the inactivation of its catalytic activity for the copper‐catalyzed alkyne‐azide cycloaddition (CuAAC) reaction.^[11]^ However, activation of the triggers by the appropriate external chemical stimuli generates the dianiline intermediate **2** that undergoes a spontaneous sequence of 1,4 and 1,6 eliminations, thereby producing the non‐interlocked Cu^I^ complex **3**. As a result of the cleavage of the mechanical bond, the Cu^I^ becomes then accessible to alkyne and azide reactants,^[12]^ hence restoring its ability to catalyze the CuAAC reaction. Our study shows that stimuli‐responsive catenane‐based catalysts can be involved in signal amplification processes,^[13]^ allowing the detection of some analytes at concentrations as low as 1 ppb.

**Figure 1:**
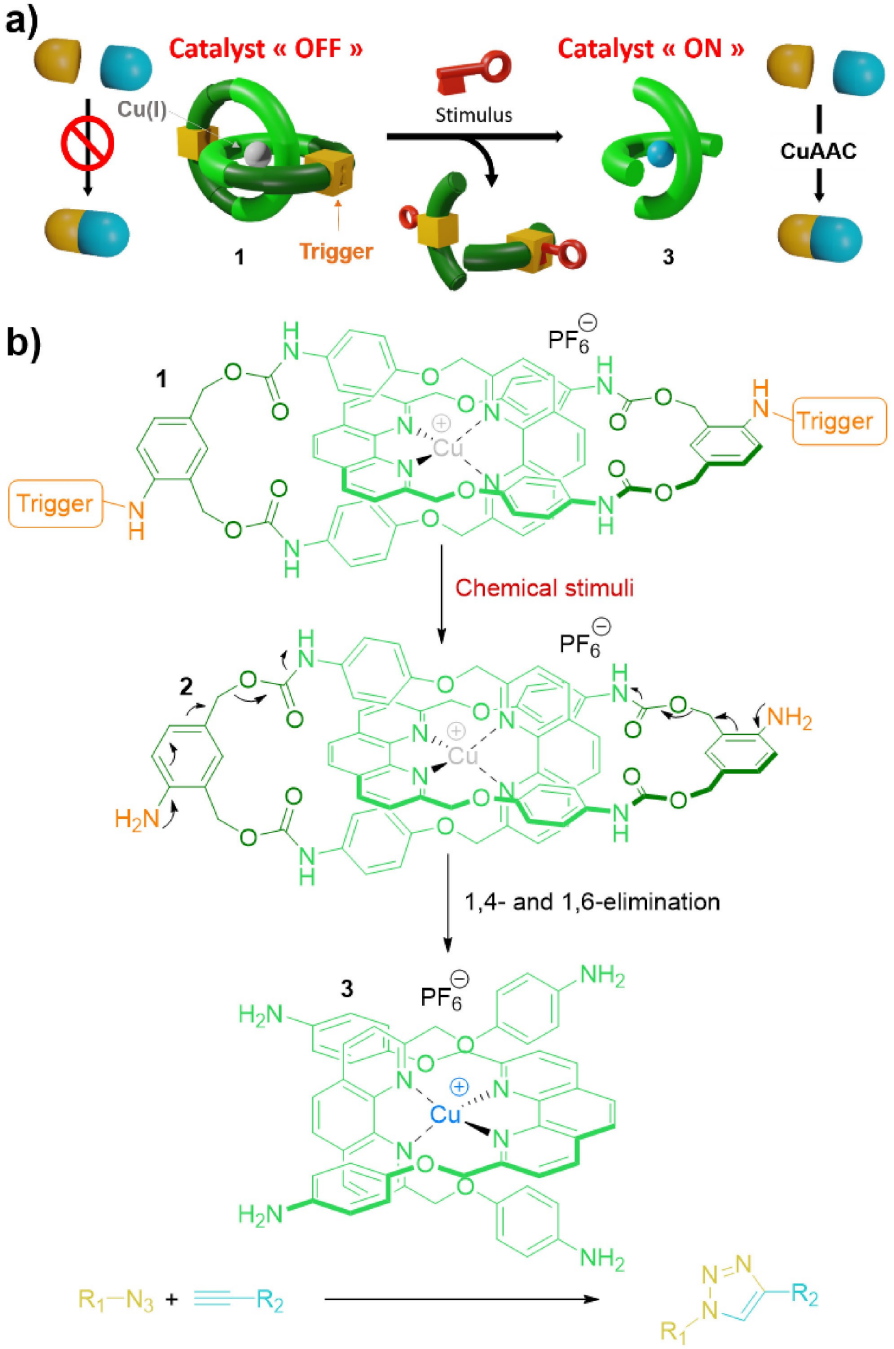
a) Action mode of the OFF/ON Cu^I^‐complexed [2]catenane catalysts **1**: when encapsulated within the [2]catenane, the Cu^I^ is inactive; an appropriate chemical stimulus triggers the controlled disassembly of the interlocked architecture, restoring the ability of Cu^I^ to catalyze the CuAAC reaction. b) Mechanism of the breakdown of the mechanical bond.

The key step in the synthesis of **1** relies on the construction of the interlocked architecture via the Cu^I^‐directed passive metal template strategy (Scheme 1).^[9]^ Thus, the Cu^I^ complex **3** was first prepared from the phenanthroline derivative **4** and Cu(MeCN)_4_PF_6_ placed for two hours in acetonitrile at 40 °C. The tetraaniline **3** reacted then with the biscarbonates **5 a** or **5 b**,^[14]^ bearing fluorenylmethoxycarbonyl‐ (Fmoc) or allyloxycarbonyl‐ (Alloc) triggers, respectively, affording the corresponding MIMs **1 a** or **1 b** (for the synthesis of precursors **4** and **5 a** see the Supporting Information). It is worth mentioning that this straightforward synthetic strategy should allow versatile access to a wide range of stimuli‐responsive [2]catenanes containing various trigger types (redox‐, light‐, enzyme‐sensitive…).

**Scheme 1:**
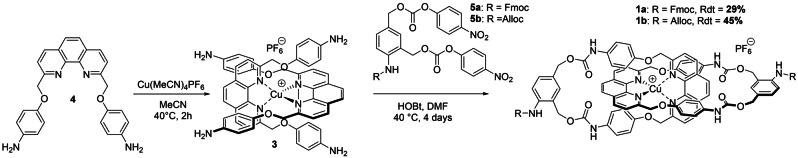
Synthesis of the stimuli‐responsive [2]catananes **1 a** and **1 b**.

We next investigated the mechanism of self‐decomposition of the [2]catenanes **1** in the presence of the appropriate chemical stimuli. For this purpose, piperidine (5 equiv) was added to a solution of the Fmoc‐protected [2]catenane **1 a** in EtOH/DMSO 8/2 at 60 °C and evolution of the mixture over time was monitored by HPLC/HRMS (Figure 2a).

**Figure 2:**
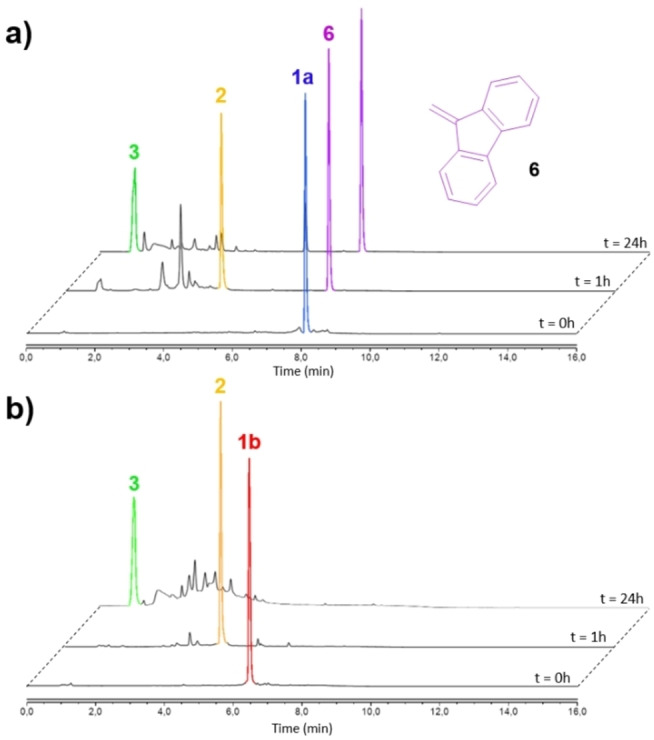
HPLC traces of [2]catenanes a) **1 a** and b) **1 b** over time. Retention times (min): **1 a**: 8.1, **1 b**: 6.5, **2**: 4.5, **3**: 1.0, **6**: 7.6.

The chromatogram showed the rapid disappearance of **1 a** (*t*=1 hour) and the emergence of peaks corresponding to the [2]catenane **2** (*m*/*z* 1317.3485 for the empirical formula C_72_H_58_CuN_10_O_12_ [*M*−PF_6_]^+^) and the 9‐methylene‐9*H*‐fluorene **6** resulting from the piperidine‐mediated cleavage of the Fmoc protecting groups. The formation of other unidentified products, probably arising from the decomposition of the self‐immolative linkers through 1,4 and 1,6 eliminations,^[15]^ was also observed. After 24 hours under these conditions, the intermediate **2** was fully converted into the Cu^I^ complex **3**, hence demonstrating that the disassembly of the interlocked molecular architecture **1 a** proceeded through the mechanism depicted on Figure 1.

We conducted a similar experiment with the Alloc‐protected [2]catenane **1 b** in the presence of Pd(PPh_3_)_4_ (0.01 equiv) and aniline (5 equiv). Once again, cleavage of the triggers led to formation of the interlocked derivative **2** (*t*=1 hour) which then self‐disassembled to yield compound **3** (*t*=24 hours, Figure 2b). These results suggest that the Cu^I^ complex **3** could be generated from the controlled decomposition of various stimuli‐responsive [2]catenanes **1** bearing different kinds of triggers, therefore offering the opportunity to design a wide range of such OFF/ON catalysts.

We then ascertained whether encapsulation of the Cu^I^ within [2]catenanes **1** inhibited its ability to catalyze the CuAAC reaction. Thus, each catenane **1** was incubated with the alkyne **7** (10 equiv) and the azide **8** (12 equiv) in EtOH/DMSO 8/2 at 60 °C, and the composition of the mixture was followed for 24 h by HPLC/HRMS (Figure 3a). As observed when the reaction was conducted without catalyst (entry 1), the formation of the triazole **9** was not detected under these conditions, neither with **1 a** nor **1 b** (entries 2 and 3, respectively). On the other hand, the phenanthroline‐derived Cu^I^ complex **3** readily catalyzed the CuAAC reaction leading to the clicked product **9** in nearly quantitative yield (entry 4). These experiments demonstrate that the mechanical bond present in catenanes **1** fully masks the copper catalyst by preventing its accessibility to the reactants of the click reaction. In contrast, the non‐interlocked Cu^I^ derivative **3**, which can dissociate, catalyzes efficiently the cycloaddition between the alkyne **7** and the azide **8**. Under these circumstances, the controlled conversion of **1** into **3** through the removal of the mechanical bond should permit activating “on‐demand” the Cu^I^ catalyst to form the triazole **9**. This hypothesis was verified by adding piperidine (0.05 equiv per Fmoc group) in the solution containing the catenane **1 a**, the alkyne **7** and the azide **8** (entry 5). The monitoring of the mixture by HPLC/HRMS showed rapid appearance of the triazole **9** concomitantly with that of complex **3** resulting from the decomposition of the [2]catenane **2** (Figure 3b, *t*=2 hours). After 24 hours of reaction, the starting compounds **7** and **8** reacted almost completely to yield **9**, thereby providing evidence of the efficient copper catalyst activation.

**Figure 3:**
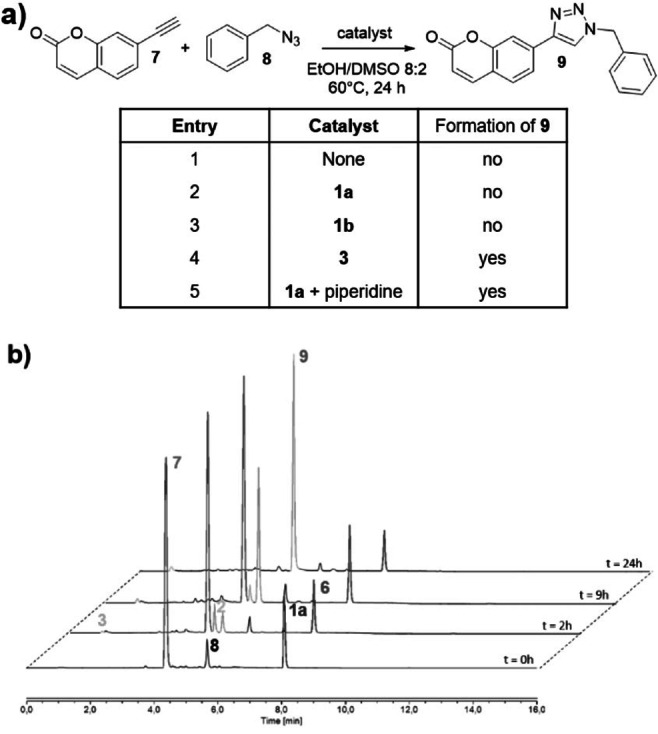
a) Formation of the triazole **9** from the alkyne **7** and the azide **8** with or without catalyst. b) HPLC traces of the CuAAC reaction conducted in the presence of the Fmoc‐protected [2]catenane **1 a** and piperidine (0.1 equiv). Retention times (min): **1 a**: 8.1, **2**: 4.5, **3**: 1.0, **6**: 7.6, **7**: 4.3, **8**: 5.6, **9**: 4.7.

To investigate the process of Cu^I^ catalyst activation in more detail, we synthesized the pseudorotaxane **11** from the macrocycle **10** and the phenanthroline derivative **4** in the presence of Cu(MeCN)_4_PF_6_ (Scheme 2). Indeed, this supramolecular assembly that can be generated in the reaction mixture by the self‐decomposition of only one macrocycle from the parent catenane, could be catalytically active to produce the triazole **9**. Thus, when **11** was incubated with the alkyne **7** (10 equiv) and the azide **8** (12 equiv) for 24 h at 60 °C, the formation of a slight amount of **9** was observed by HPLC (see the Supporting Information). This result indicated that the cleavage of only one macrocycle was sufficient to switch on the catalytic activity of Cu^I^. However, since the CuAAC reaction reached completion under the same conditions with complex **3**, the pseudorotaxane **11** appeared far less efficient to catalyze the formation of the triazole **9**.

**Scheme 2:**
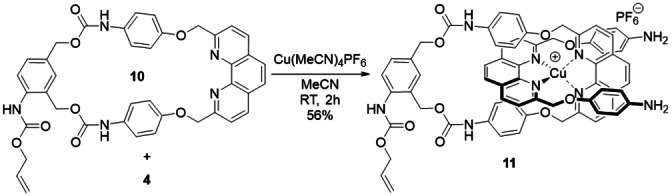
Synthesis of the pseudorotaxane **11**.

We also evaluated the stability of the [2]catenane **1 b** in the presence of cyanide ions in order to demonstrate that the catenand effect^[16]^ was responsible for Cu^I^ inhibition to catalyze the CuAAC reaction. For this purpose, **1 b** was incubated with increasing concentrations of KCN and the formation of the copper free catenane **1 c** was monitored by UPLC‐HRMS (Figure 4). These experiments allowed the dissociation constants (*K*_d_) for **1 b** to be determined as a function of cyanide ion concentration. Thus, when **1 b** was dissolved in CH_3_CN/H_2_O (9 : 1) in the absence of KCN, the copper‐free catenane **1 c** was not observed in the mixture by UPLC‐HRMS (Figure 4c). In contrast, addition of KCN induced demetallation of **1 b**, leading to the formation of **1 c**. These experiments highlighted the higher stability of the [2]catenane **1 b** compared to copper phenanthroline complexes such as **3**. Indeed, the dissociation constant for **1 b** was largely superior than those reported in the literature for various bis(1,10‐phenanthroline)copper(I) (p*K*_d_=5.5–7.0).^[17]^ In order to reach such dissociation constant values, **1 b** has to be incubated in the presence of a large excess of cyanide ion (p*K*_d_=6.7 with 50 000 equiv of KCN). These results confirmed that the inhibition of Cu^I^ to catalyze the CuAAC reaction within the structure of **1 b** was the consequence of the catenand effect.

**Figure 4:**
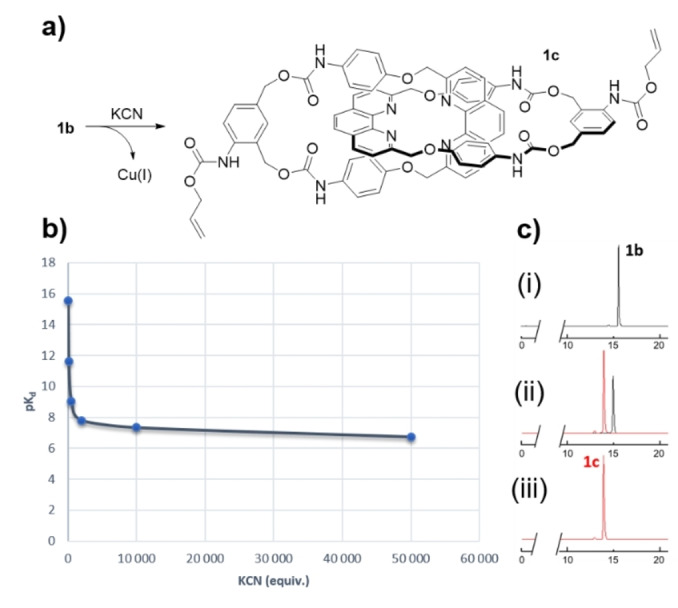
a) Synthesis of the [2]catenane **1 c** through demetallation of **1 b**. b) p*K*_d_ values as a function of KCN equivalents. c) Extracted ion chromatograms for **1 b** and **1 c** in the presence of (i) 0 equiv KCN; (ii) 10 000 equiv KCN; (ii) 50 000 equiv KCN.

Interestingly, Au‐Yeung reported recently that phenanthroline‐derived copper catenanes were more efficient than the corresponding bis(1,10‐phenanthroline)copper(I) complexes for catalyzing the cross‐dehydrogenative C−O coupling of phenols and bromodicarbonyls.^[18]^ Therefore, it appears that the catenand effect allows the versatile modulation of Cu^I^ catalytic activity in various chemical reactions.

Interestingly, the production of the triazole **9** in the presence of the [2]catenane **1 a** and piperidine was the result of a double‐catalyst cascade. Indeed, in this case, full activation of the [2]catenane was achieved with a catalytic amount of piperidine^[19]^ (0.05 equiv per Fmoc group). Furthermore, the subsequent CuAAC reaction proceeded with 0.1 equivalent of the Cu^I^ catalyst compared to the quantities of starting materials **7** and **8** introduced in the mixture. Such a catalyst combination, in which the first catalytic cycle acts as an activator for the second catalytic cycle, leads to an amplification process that can be useful for sensing purposes.

In this context, we postulated that the Alloc‐protected [2]catenane **1 b** could be a powerful tool for the detection of low palladium concentrations, since its activation to produce triazole **9** involves a sequence of two metal catalysts. In order to investigate this sensing strategy, we developed the experimental procedure depicted on Figure 5a. Thus, various quantities of Pd(PPh_3_)_4_ were added to a solution of **1 b** and aniline (step 1). The mixture was stirred for 12 hours at 60 °C, then the coumarine **7** and the azide **8** were incubated in the reaction medium (step 2). After 24 hours, the mixture was analyzed by HPLC/UV to detect the triazole **9** as a reporter of the presence of Pd^0^ in the solution (step 3).

**Figure 5:**
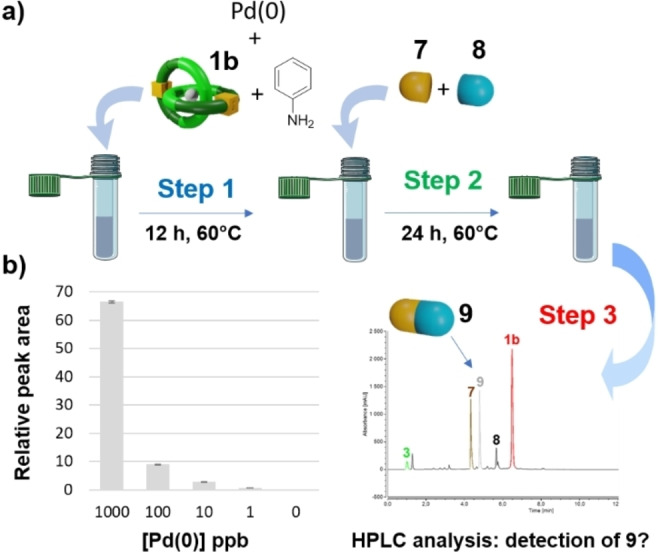
a) Overview of the Pd^0^‐sensing procedure. Step 1: conditional activation of the catalyst **1 b** in the presence of Pd^0^ leading to the formation of the non‐interlocked Cu^I^ complex **3**. Step 2: conditional production of the triazole **9** from the alkyne **7** and the azide **8** via the CuAAC reaction. Step 3; HPLC/UV analysis of the reaction mixture to detect the presence of triazole **9**. b) Relative peak area of compound **9** recorded by HPLC/UV as a function of the quantity of Pd^0^ introduced in the reaction medium (experiments were carried out in triplicate).

As shown in Figure 5b, when palladium was introduced in the mixture at concentrations ranging from 1000 to 1 ppb, the formation of **9** was clearly detected by HPLC/UV (limit of detection below 1 ppb, see the Supporting Information). The sensing procedure was also conducted without Pd^0^. In this case, no trace of **9** was observed, therefore indicating that the palladium was necessary to trigger the CuAAC reaction. Overall, these results demonstrate that the Alloc‐protected [2]catenane **1 b** is an efficient OFF/ON catalyst, allowing the sensing of low palladium concentrations via a double‐catalyst amplification methodology.

In summary, we developed stimuli‐responsive [2]catenane‐based catalysts that can be activated “on‐demand” through the controlled breakdown of the mechanical bond. These MIMs that contain self‐opening macrocycles are readily accessible by a straightforward and versatile synthetic strategy that enables the preparation of a wide range of such OFF/ON catalysts. Thus, our approach offers the opportunity to design diverse masked Cu^I^ catalysts that could be triggered under various chemical, biochemical or physical conditions. These stimuli‐responsive [2]catenanes can be involved in a signal amplification process initiated by either a metal or an organic catalyst. We showed that this double‐catalyst amplification can be useful for the sensing of some analytes such as Pd^0^ at low concentrations. This proof of principle opens a new field of investigations for MIMs with the design of OFF/ON metal catalysts based on the disassembly of the interlocked molecular architecture.

## Conflict of interest

The authors declare no conflict of interest.

## Supporting information

As a service to our authors and readers, this journal provides supporting information supplied by the authors. Such materials are peer reviewed and may be re‐organized for online delivery, but are not copy‐edited or typeset. Technical support issues arising from supporting information (other than missing files) should be addressed to the authors.

Supporting Information

## Data Availability

The data that support the findings of this study are available from the corresponding author upon reasonable request.
