# Supplementary material for: Stimuli‐Responsive Catenane‐Based Catalysts
Source: Angew Chem Int Ed Engl. 2022 Dec 28;62(6):e202216787. doi: 10.1002/anie.202216787 (PMC10107136; doi:10.1002/anie.202216787)
Supplement: Supplementary file 1 — Supporting Information [file ANIE-62-0-s001.pdf]

Angewandte Chemie
Eine Zeitschrift der Gesellschaft Deutscher Chemiker

Supporting Information

Stimuli-Responsive Catenane-Based Catalysts

A. Bessaguet, Q. Blancart-Remaury, P. Poinot, I. Opalinski, S. Papot**

Supporting Information

Table of Contents

I. CHEMISTRY SECTION	S2
I.1. General experimental methods	S2
I.2. Synthetic schemes towards compounds 5a, 4, 3, 1a, 1b, 10, 11	S3
I.3. Synthetic procedures and characterization details with ^{1}H NMR, ^{13}C NMR and ^{19}F NMR plots	S5
II. STUDY OF CATENANES 1a AND 1b	S26
II.1. Stability of catenanes 1a and 1b	S26
II.2. HPLC and UPLC-HRMS decomposition of catenanes 1a and 1b	S27
II.3. Catalytic activity of compound 3	S31
II.4. Catalytic activity of compound 11	S31
II.5. Activation of catenane 1a	S32
II.6. Activation of catenane 1b	S34
II.7. Catalytic detection of Pd^0	S35
II.8. Determination of the dissociation constante (pKd) of catenane 1b	S36
III. REFERENCES	S38

I. Chemistry Section

I.1. General experimental methods

Synthesis: All reactions were performed under an argon atmosphere. Unless otherwise stated, solvents used were of HPLC quality. For oxygen sensitive reactions like copper catalysed reaction, solvents were deoxygenated by purging with argon. Chemicals were of analytical grade from commercial sources and were used without further purification. Reaction were monitored using precoated silica gel TLC plates Macherey-Nagel ALUGRAM® SIL G/UV_{254}. (0.2 mm silica gel 60). Spots were visualized under 254 nm UV light and/or by dipping the TLC plate into a solution of phosphomolybdic acid (3 g) in ethanol (100 mL) followed by heating with a heat gun. Automatic flash chromatography was performed on Combi Flash 210i (Teledyne-Isco) with pre-packed column purchased from Interchim, Silicycle or Buchi. Particles size (from 50 µm to 15 µm) and column size (4 g to 280 g) were adapted according the difficulty of the purification and the quantity of crude product.

Analysis: ^{1}H, ^{13}C and ^{19}F NMR spectra were respectively recorded at 400 MHz, 100MHz and 376 MHz, on a Bruker 400 Avance III instrument, equipped with an ultrashielded plus magnet and a BBFO 5 mm broadband probe or at 500 MHz, 126 MHz, and 470 MHz on a Bruker 500 Avance NEO instrument, equipped with an ultrashielded magnet and a Prodigy cryoprobe. Chemical shifts (δ) are reported in parts per million (ppm) from low to high field and referenced to residual solvent peaks or using C_6F_6 as external reference for ^{19}F. Coupling constants (*J*) are reported in hertz (Hz). Standard abbreviations indicating multiplicity are used as follows: b = broad, s = singlet, d = doublet, t = triplet, q = quartet, qi = quintet, m = multiplet, dd = doublet of doublets. High-resolution mass spectra (HRMS) were performed on a Bruker maXis mass spectrometer by the "Fédération de Recherche" ICOA/CBM (FR2708) platform and by the mass spectrometry service or Poitiers (Platina) on a LC-QTof MaXis Impact, Bruker.

UPLC-HRMS method : Reaction follow-up were performed on LC-QExactive orbitrap (Thermo Fisher) with Kinetec (1.7u F5 100 x 2.1 mm) column in a thermostatically controlled oven at 30 °C. The mass range was from 400 to 2500 m/z in positive mode with a resolution of 70 000 and capillary voltage of 4kV and capillary temperature of 270 °C. Eluents were A (H_2O + 0.1 % formic acid), B (MeCN + 0.1 % formic acid) and solvent flow: 0.3 $mL.min^{-1}$. Method: gradient elution beginning with A/B 60:40 for 2 minutes then reaching A/B 0:100 within 18 minutes, then isocratic A/B 0:100 for 5 minutes and linear gradient toward A/B 60:40 within 2 minutes.

HPLC method: Reaction follow-up and compound purity were performed on DIONEX Ultimate 3000 with UV light set to 254 nm with MACHEREY-NAGEL Nucleoshell®(150/4.6, RP18, 5 µm) column in a thermostatically controlled oven at 30 °C. Spectra analysis was carried out with the software Chromeleon. Eluents were A (H_2O + 0.2 % TFA), B (MeCN) and solvent flow: 1.25 mL.min^{-1}. Method: linear gradient beginning with A/B 80:20 reaching A/B 0:100 within 8 minutes, then isocratic A/B 0:100 for 6.5 minutes and linear gradient toward A/B 80:20 within 1.5 minutes.

The following compounds were synthesized according to literature procedures:

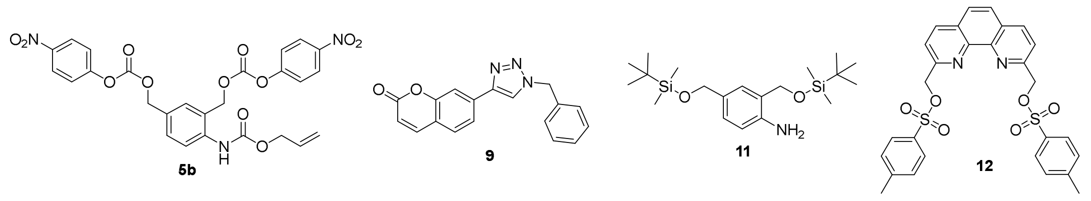

Scheme S1. *References of literature procedures:* compound **5b**,[1] compound **9**,[2] compound **11**,[3] compound **12**.[4]

I.2. Synthetic schemes towards compounds 5a, 4, 3, 1a, 1b, 10, 11.

Compound **5a** was prepared according to the following strategy:

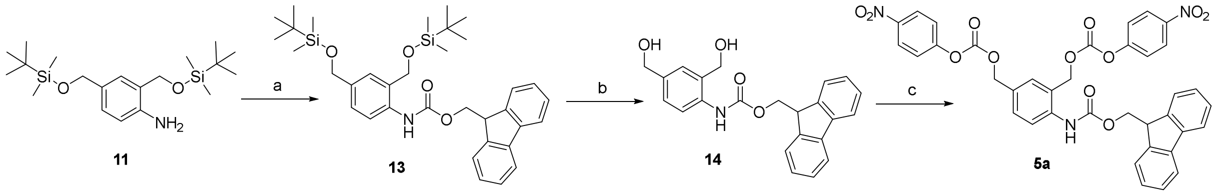

Scheme S2. *Reagents and conditions:* (a) 9-fluorenylmethoxycarbonyl chloride, pyridine, DCM, 0 °C to RT, 24 h, 92%; (b) *p*-toluenesulfonic acid monohydrate (APTS.H_2O), THF, H_2O, 0 °C to RT, 24 h, not purified; (c) 4-nitrophenyl chloroformate, pyridine, THF, 0 °C to RT, 24 h, 48% (two steps).

Compound **4** was prepared according to the following strategy:

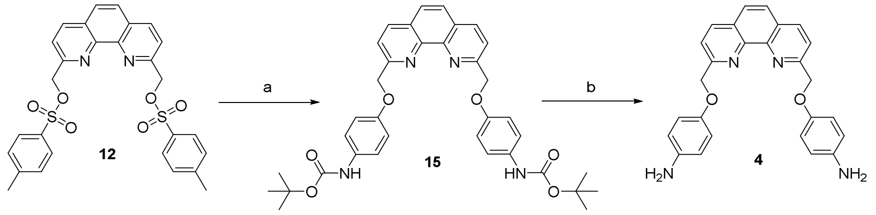

Scheme S3. *Reagents and conditions:* (a) *N*-Boc-4-hydroxyaniline, K_2CO_3, MeCN/Toluene 4/1, reflux, 24 h, not purified; (b) TFA, DCM, RT, 2 h, 81% (two steps).

Compound **1a** was prepared according to the following strategy:

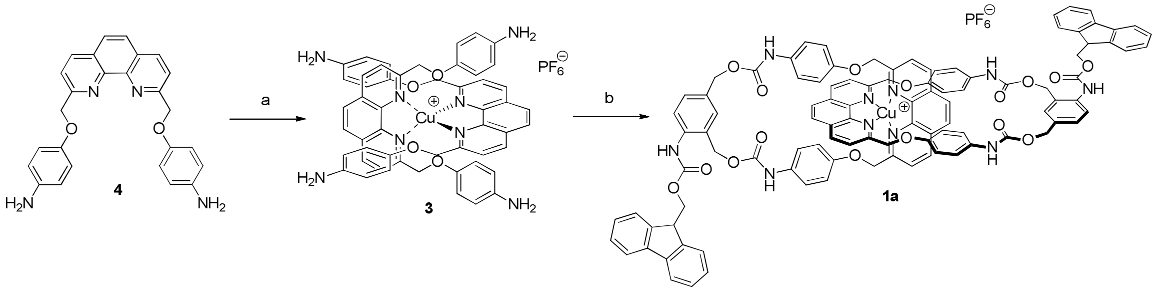

Scheme S4. *Reagents and conditions:* (a) $[Cu(MeCN)_4]PF_6$, MeCN, 40 °C, 2 h, quant.; (b) **5a**, HOBt, DMF, 40 °C, 4 days, 29%.

Compound **1b** was prepared according to the following strategy:

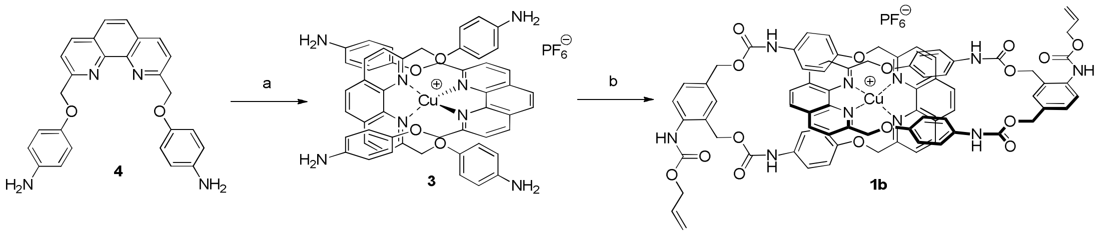

Scheme S5. *Reagents and conditions:* (a) $[Cu(MeCN)_4]PF_6$, MeCN, 40 °C, 2 h, quant.; (b) **5b**, HOBt, DMF, 40 °C, 4 days, 45%.

Compound **11** was prepared according to the following strategy:

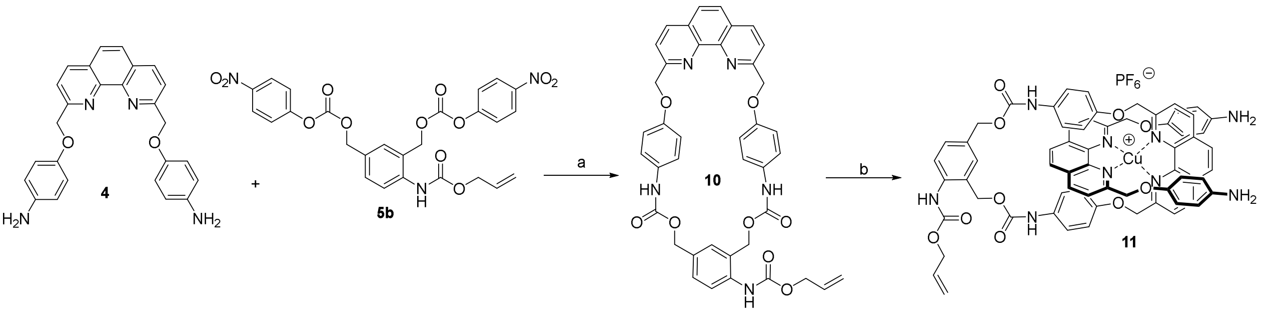

Scheme S6. *Reagents and conditions:* (a) HOBt, DMF, 30 °C, 4 days, 36%; (b) **4**, $[Cu(MeCN)_4]PF_6$, MeCN, RT, 2 h, 56%

I.3. Synthetic procedures and characterization details with ^{1}H NMR, ^{13}C NMR and ^{19}F NMR plots

Preparation of compound 13

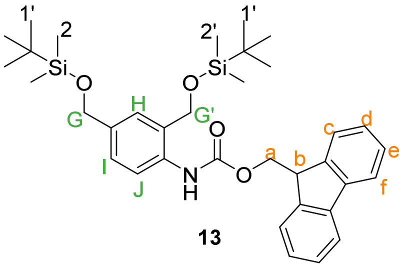

13

To a solution of **11** (872 mg, 2.28 mmol, 1.0 equiv.) and pyridine (221 µL, 2.74 mmol, 1.2 equiv.) in DCM (1.5 mL) at 0 °C was added dropwise a solution of 9-fluorenylmethoxycarbonyl chloride (649 mg, 2.51 mmol, 1.1 equiv.) in DCM (1.5 mL). The mixture was stirred for 18 hours at room temperature before $HCl_{(aq)}$ (1M) (5 mL) was added. Layers were separated and aqueous layer was extracted with DCM (2 x 5 mL). Combined organic layer were washed with brine (5 mL), dried over $MgSO_4$ and concentrated *in vacuo*. Purification by column chromatography over silica gel (PE/EtOAc, 95:5, R_f = 0.6 (90:10)) afforded compound **13** (1.27 g, 2.10 mmol, 92%) as a colorless oil.

^{1}H NMR (400 MHz, $CDCl_3$) δ 8.42 (bs, 1H, $H_{ArNHCO\ carbamate}$), 8.01 (bs, 1H, H_J), 7.80 (d, 2H, *J* = 7.5, H_f), 7.67 (d, 2H, *J* = 7.4, H_c), 7.43 (t, 2H, *J* = 7.5, H_e), 7.34 (td, 2H, *J* = 7.4, 1.1, H_d), 7.27 (d, 1H, *J* = 8.0, H_I), 7.11 (d, 1H, *J* = 1.7, H_H), 4.77-4.71 (2s, 4H, $H_{G\text{-}G'}$), 4.54 (d, 2H, *J* = 7.0, H_a), 4.31 (t, 1H, *J* = 7.0, H_b), 0.97-0.95 (2s, 18H, $H_{1\text{-}1'}$), 0.13-0.12 (2s, 12H, $H_{2\text{-}2'}$).

^{13}C NMR (100 MHz, $CDCl_3$) δ 153.8 (C_{NHCOO}), 144.0-141.5-136.9-136.1-128.4 ($C_{quat.\ arom.}$), 127.8 (C_e), 127.2 (C_d), 126.7 (C_I), 126.1 (C_H), 125.2 (C_c), 120.1 ($C_{f\text{-}J}$), 67.0 (C_a), 65.6-64.7 ($C_{G\text{-}G'}$), 47.3 (C_b), 26.1-25.9 ($C_{1\text{-}1'}$), 18.5-18.2 ($C_{quat.\ t\text{-}But.}$), -5.1 - -5.3 ($C_{2\text{-}2'}$).

HRMS (ESI^{+}) *m/z* = 604.3264 $[M+H]^+$ (calc. for $C_{35}H_{50}NO_4Si_2$: 604.3273 $[M+H]^+$).

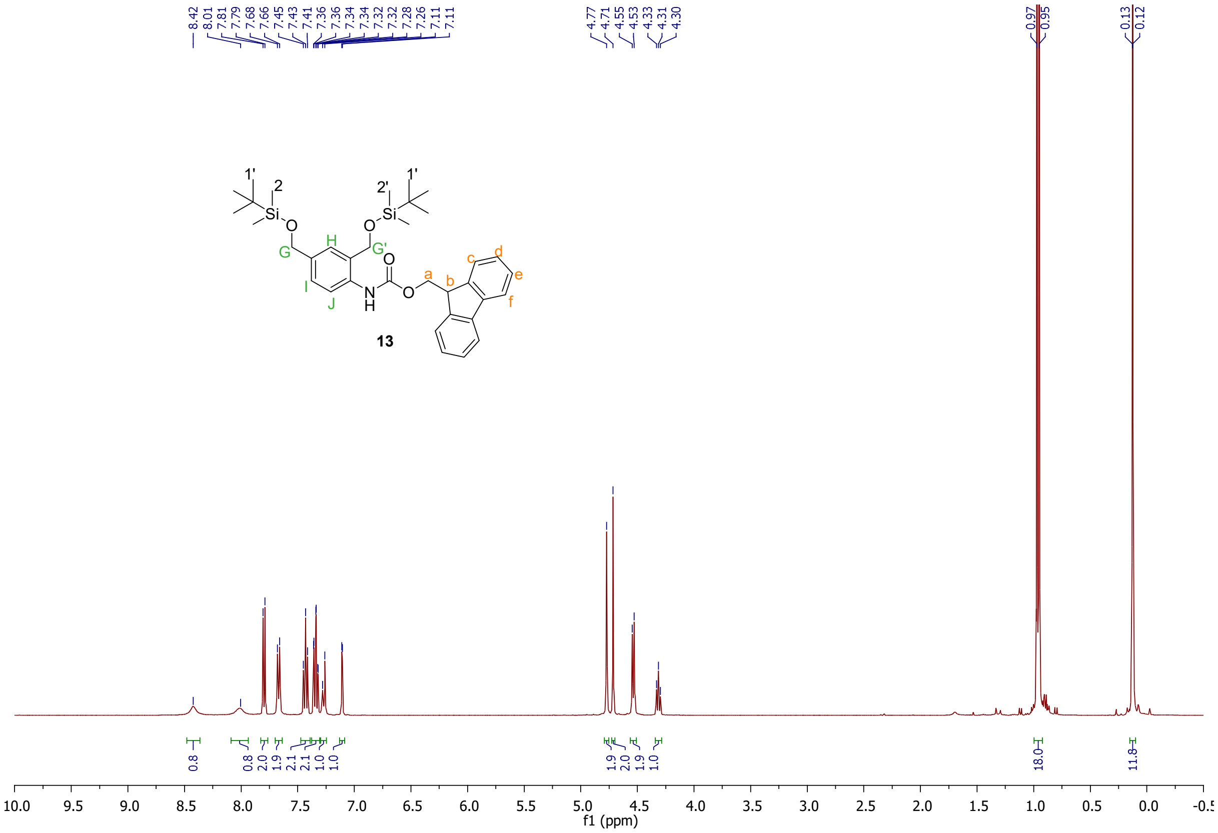

^{1}H NMR spectrum of **13**, 400 MHz, 298 K, $CDCl_3$

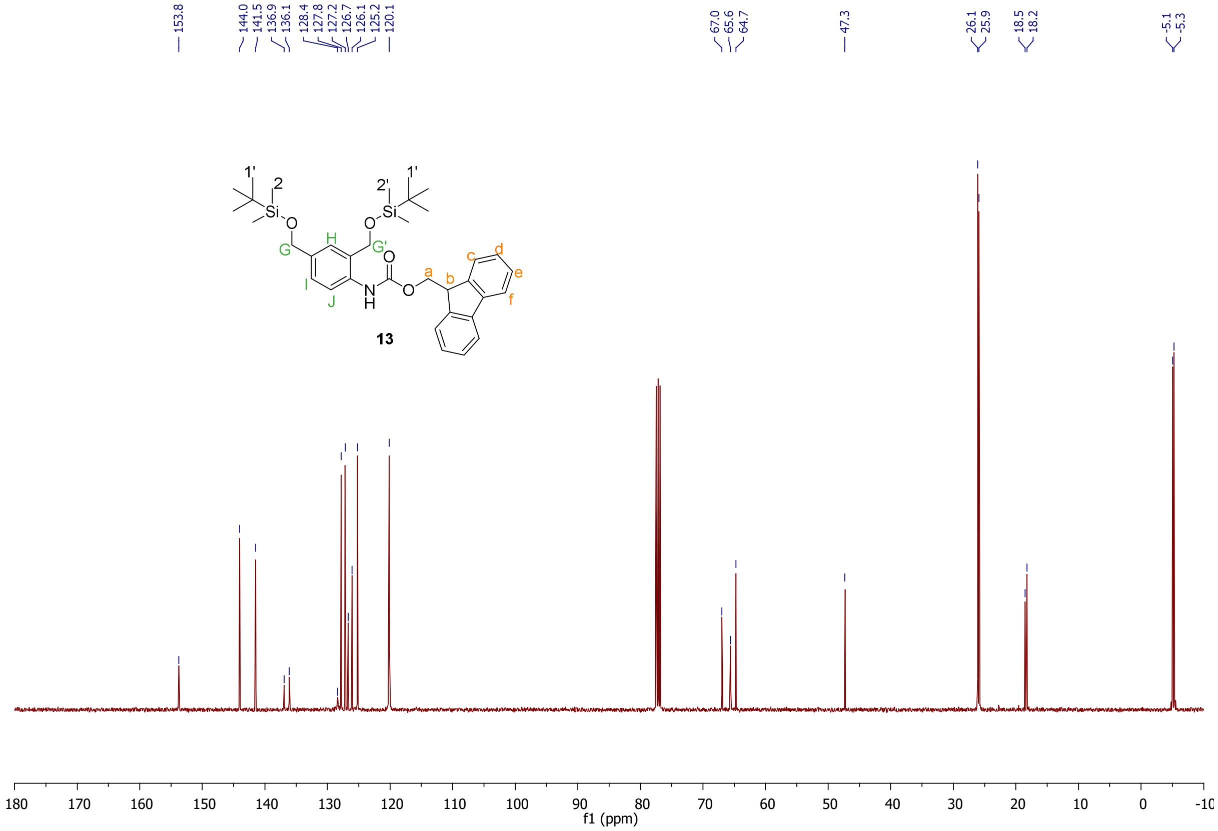

^{13}C NMR spectrum of **13**, 100 MHz, 298 K, $CDCl_3$

Preparation of compound 5a

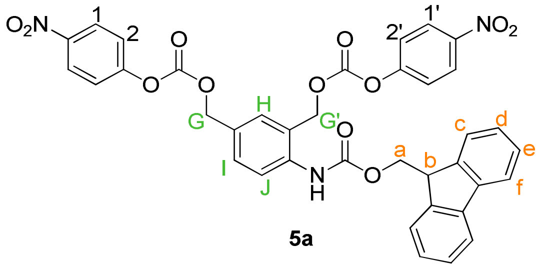

5a

To a stirred solution of compound **13** (1.35 g, 2.24 mmol, 1.0 equiv.) in THF (18 mL) cooled at 0 °C, was added dropwise a solution of *p*-toluenesulfonic acid monohydrate (APTS.H_2O) (128 mg, 671 µmol, 0.3 equiv.) in water (2.0 mL). The reaction mixture was stirred at room temperature for 24 hours. The reaction was hydrolyzed with H_2O and the solution was extracted with Et_2O (2 x 10 mL). The aqueous layer was saturated with solid NaCl and extracted with Et_2O (2 x 10 mL)). The combined organic layers were dried over $MgSO_4$, filtered and concentrated *in vacuo* to give the crude di-alcohol **14** without further purification as a white solid (550 mg, 65%).

To a stirred solution of 4-nitrophenyl chloroformate (362 mg, 1.80 mmol, 3.0 equiv.) in anhydrous THF (15 mL) cooled at 0 °C, was added dropwise pyridine (150 µL, 1.80 mmol, 3.0 equiv.). The reaction mixture was stirred for 20 minutes at 0 °C and the crude di-alcohol (225 mg, 600 µmol, 1.0 equiv.) was added. The mixture was warmed up to room temperature and stirred for 18 hours. The reaction was hydrolyzed with a saturated $NaHCO_{3(aq)}$ solution and the aqueous phase was extracted with DCM (3 x 5 mL). The combined organic layers were dried over $MgSO_4$, filtered and concentrated *in vacuo*. Purification by column chromatography over silica gel (PE/EtOAc, gradient elution 90:10 to 60:40, R_f = 0.5 (60:40)) afforded compound **5a** (312 mg, 442 µmol, 74%) as a white solid.

^{1}H NMR (500 MHz, CD_2Cl_2) δ 8.26 (d, 4H, *J* = 9.2, $H_{1\text{-}1'}$), 7.79 (d, 2H, *J* = 7.6, H_f), 7.63 (d, 2H, *J* = 7.5, H_c), 7.54 (m, 2H, $H_{H\text{-}J}$), 7.49 (d, 1H, *J* = 8.2, H_I), 7.42-7.38 (m, 6H, $H_{2\text{-}2'\text{-}e}$), 7.28 (td, 2H, *J* = 1,7.5, 7.6, H_d), 5.35-5.29 (2s, 4H, $H_{G\text{-}G'}$), 4.52 (d, 2H, *J* = 6.9, H_a), 4.30 (t, 1H, *J* = 6.9, H_b).

^{13}C NMR (126 MHz, CD_2Cl_2) δ 156.0-155.8-154.2-153.5-153.0-146.1-146.0-144.3-141.8-138.1 ($C_{quat.}$), 132.3 ($C_{H\text{-}I}$), 131.4 (C_J), 128.3 (C_e), 127.6 (C_d), 125.8-125.8 ($C_{1\text{-}1'}$), 125.6 (C_c), 122.4 ($C_{2\text{-}2'}$), 120.5 (C_f), 70.6-68.3 ($C_{G\text{-}G'}$), 67.8 (C_a), 47.6 (C_b).

HRMS (ESI$^+$) *m/z* = 728.1490 $[M+Na]^+$ (calc. for $C_{37}H_{27}N_3O_{12}Na$: 728.1487 $[M+Na]^+$).

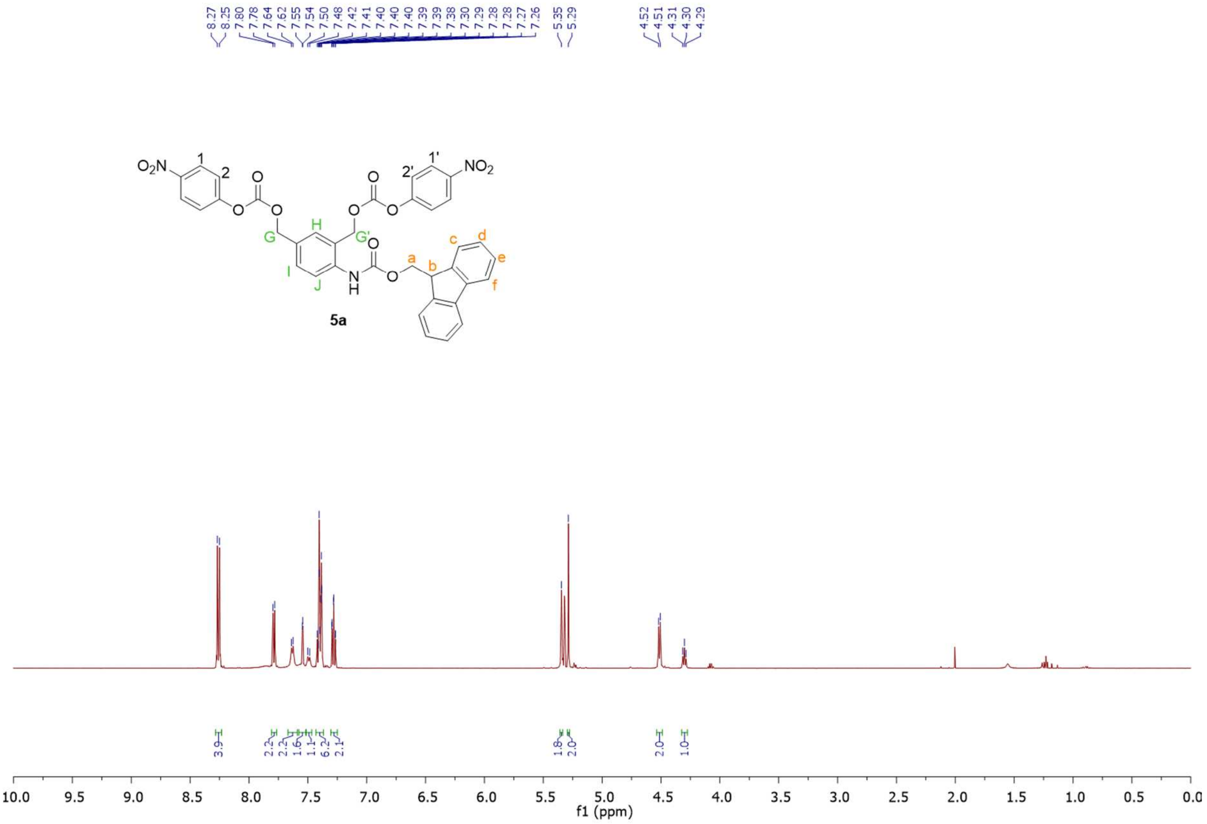

^{1}H NMR spectrum of **5a**, 500 MHz, 298 K, CD_2Cl_2

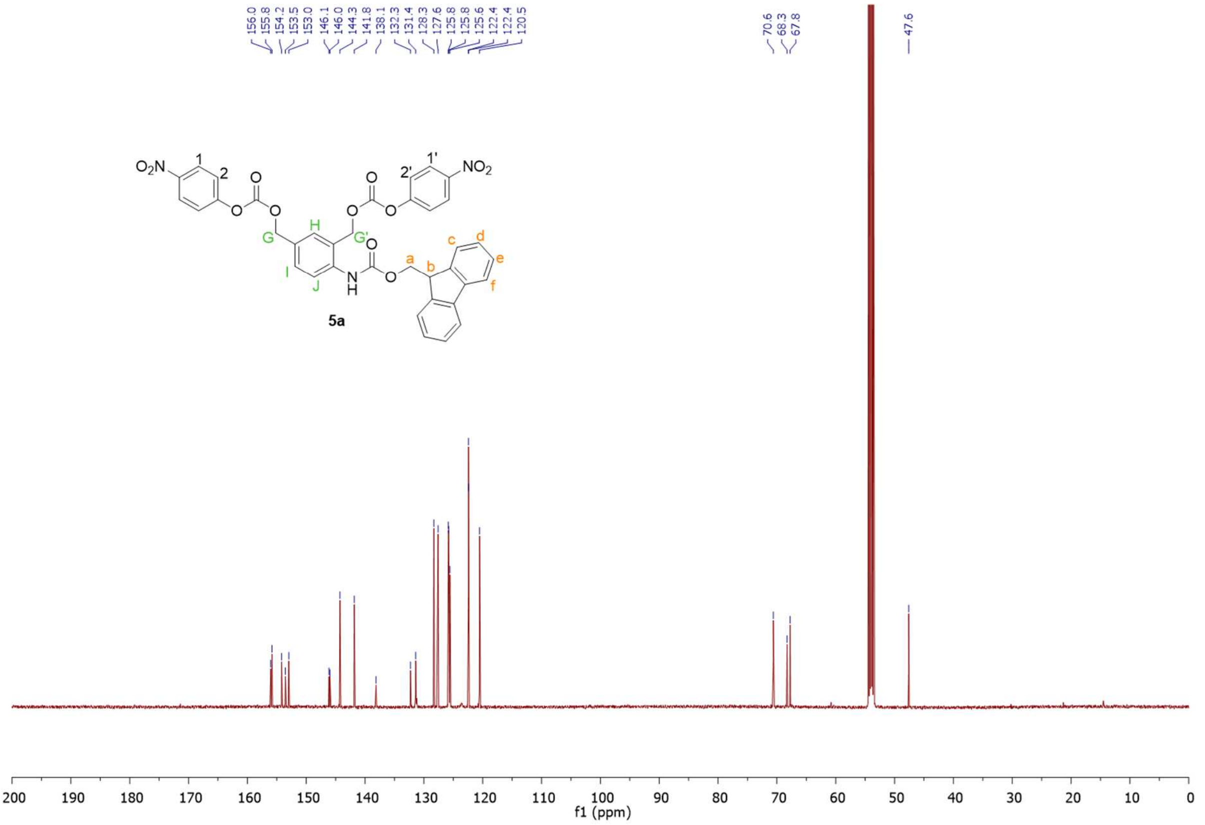

^{13}C NMR spectrum of **5a**, 126 MHz, 298 K, CD_2Cl_2

Preparation of compound 4

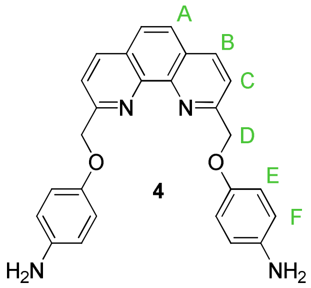

A solution of **12** (1.28 g, 2.33 mmol, 1.0 equiv.), K_2CO_3 (806 mg, 5.83 mmol, 2.5 equiv.) and *N*-Boc-4-hydroxyaniline (1.03 g, 4.9 mmol, 2.1 equiv.) in MeCN/Toluene 4:1 (30 mL) was heated to reflux for 18 hours. Solvents were removed *in vacuo*. The residue was partitioned between DCM (20 mL) and H_2O (10 mL). Layers were separated and aqueous layer was extracted with DCM (3 x 10 mL). Combined organic layers were washed with brine (10 mL), dried over $MgSO_4$ and concentrated to give the crude **15** without further purification. The crude (1.50 g, 2.41 mmol, 1.0 equiv.) was dissolved in DCM (50 mL) before TFA (738 µL, 4.0 equiv.) was added dropwise. The reaction mixture was stirred for 2 hours at room temperature. The crude mixture was diluted with H_2O (30 mL) and quenched with solid $NaHCO_3$. Layers were separated and aqueous layer was extracted with DCM (3 x 10 mL). Combined organic layers were washed with brine (10 mL), dried over $MgSO_4$ and concentrated *in vacuo*. The residue was purified by column chromatography over silica gel (DCM/MeOH gradient elution 100:0 to 95:5, R_f = 0.2 (90:10)) to obtain compound **4** (800 mg, 1.89 mmol, 81% over 2 steps) as a white solid.

^{1}H NMR (500 MHz, DMSO-*d*$_6$) δ 8.52 (d, 2H, *J* = 8.3, H_B), 7.99 (s, 2H, H_A), 7.88 (d, 2H, *J* = 8.3, H_C), 6.83 (d, 4H, *J* = 8.9, $H_{E\text{ or }F}$), 6.53 (d, 4H, *J* = 8.9, $H_{E\text{ or }F}$), 5.37 (s, 4H, H_D), 4.68 (s, 4H, H_{NH2}).
^{13}C NMR (126 MHz, DMSO-*d*$_6$) δ 158.2-149.4-144.6-142.9 ($C_{quat.}$), 137.2 (C_B), 127.9 ($C_{quat.}$), 126.4 (C_A), 121.1 (C_C), 115.7 ($C_{E\text{ or }F}$), 115.0 ($C_{E\text{ or }F}$), 71.5 (C_D).

HRMS (ESI$^+$) *m/z* = 423.1814 $[M+H]^+$ (calc. for $C_{26}H_{23}N_4O_2$: 423.1816 $[M+H]^+$).

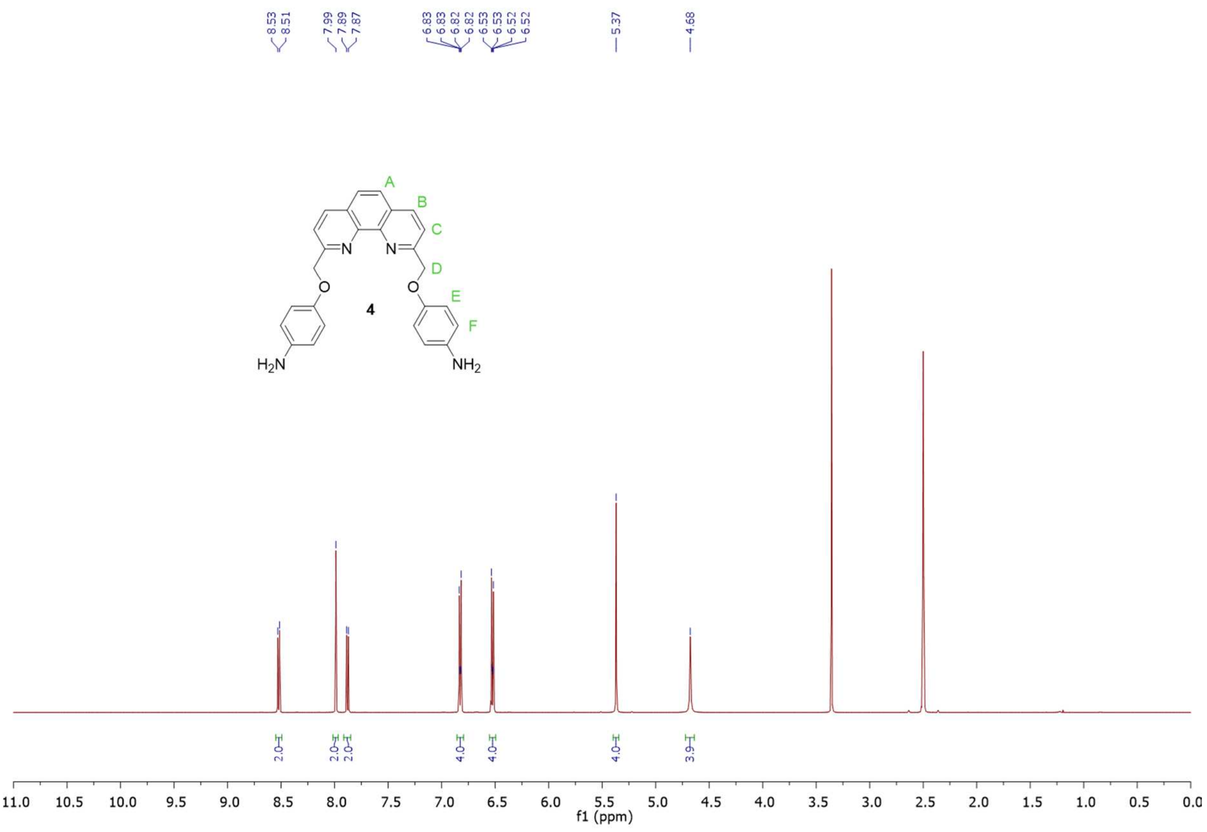

^{1}H NMR spectrum of **4**, 500 MHz, 298 K, DMSO-d_6

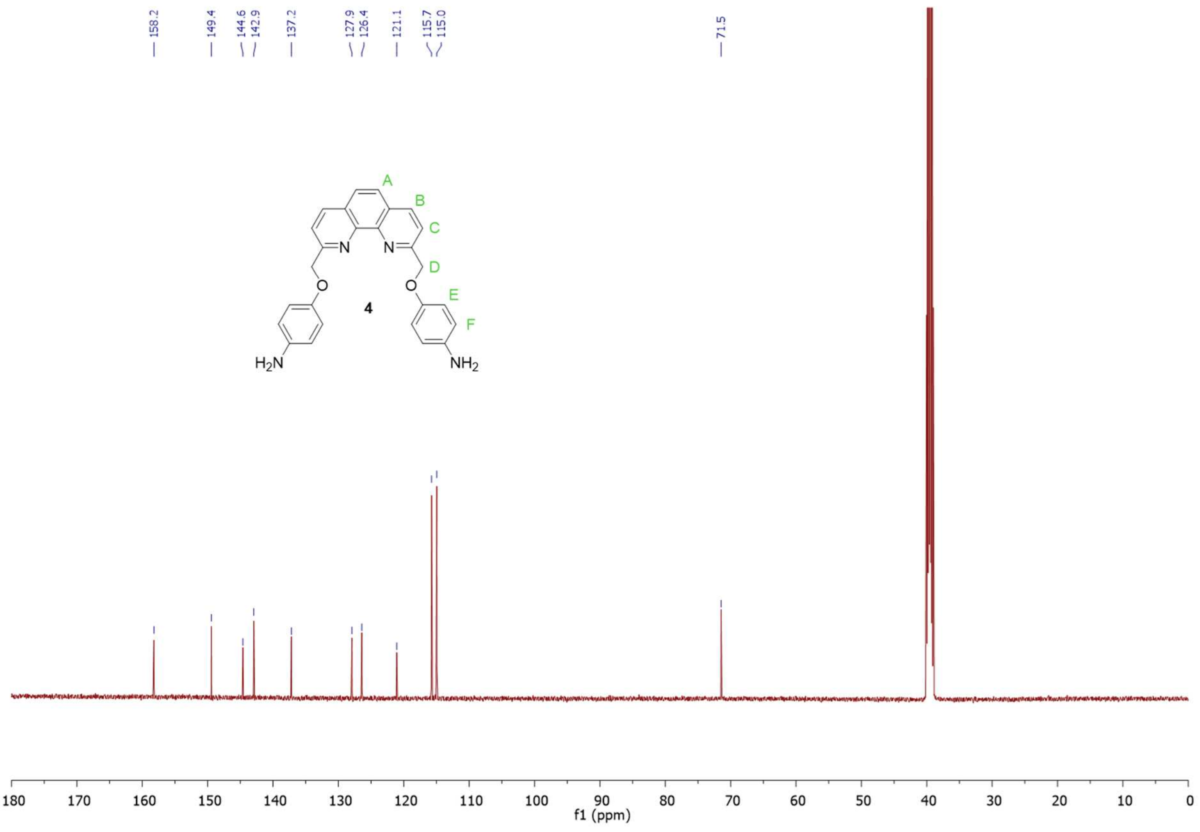

^{13}C NMR spectrum of **4**, 126 MHz, 298 K, DMSO-d_6

Preparation of compound 3

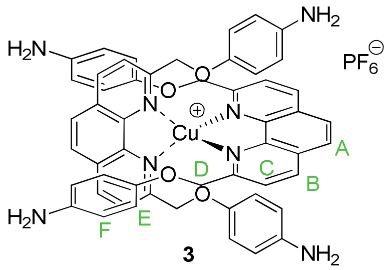

3

To a solution of **4** (50.0 mg, 118 µmol, 2.05 equiv.) in MeCN (10 mL) was added $[Cu(MeCN)_4]PF_6$ (21.5 mg, 57.7 µmol, 1.0 equiv.). The dark red solution was stirred for 2 hours at 40 °C then solvent was evaporated to afford compound **3** (60.8 mg, quant.) as a dark red solid.

^{1}H NMR (500 MHz, DMSO-*d*$_6$) δ 8.81 (d, 4H, *J* = 8.3, H_B), 8.22 (s, 4H, H_A), 8.09 (d, 4H, *J* = 8.3, H_C), 6.05 (d, 8H, *J* = 8.3 , $H_{E\ or\ F}$), 5.91 (d, 8H, *J* = 8.3, $H_{E\ or\ F}$), 4.80 (s, 8H, H_D), 4.59 (bs, 8H, H_{NH2}).
^{13}C NMR (126 MHz, DMSO-*d*$_6$) δ 169.8-154.5-146.8-140.9-140.6 ($C_{quat.}$), 136.5 (C_B), 126.9 ($C_{quat.}$), 125.0 (C_A), 122.5 (C_C), 116.5 ($C_{quat.}$), 112.8 (C_{E-F}), 69.4 (C_D).
^{19}F NMR (470 MHz, DMSO-*d*$_6$) δ -70.07 (d, *J* = 711.2, PF_6^-).

HRMS (ESI$^+$) *m/z* = 907.2776 $[M-PF_6]^+$ (calc. for $C_{52}H_{44}CuN_8O_4$: 907.2783 $[M-PF_6]^+$).

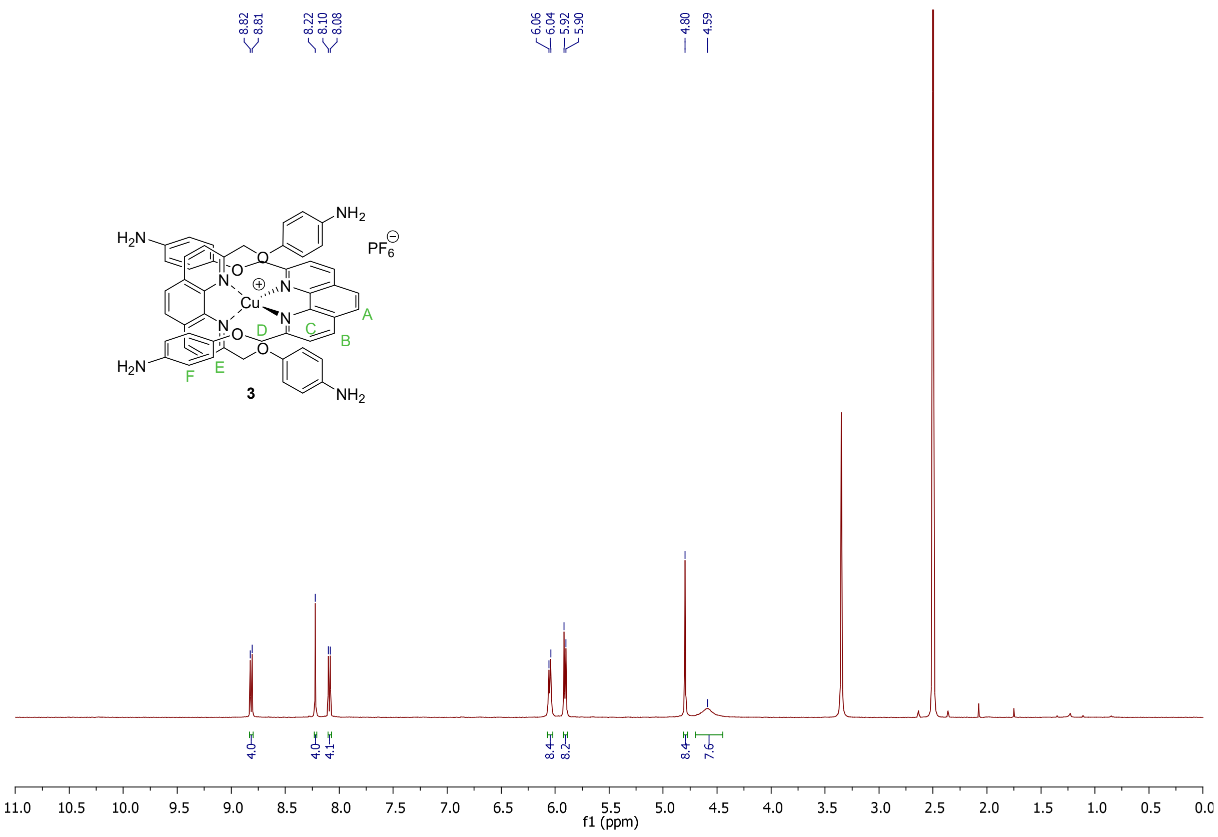

^{1}H NMR spectrum of **3**, 500 MHz, 298 K, DMSO-d_6

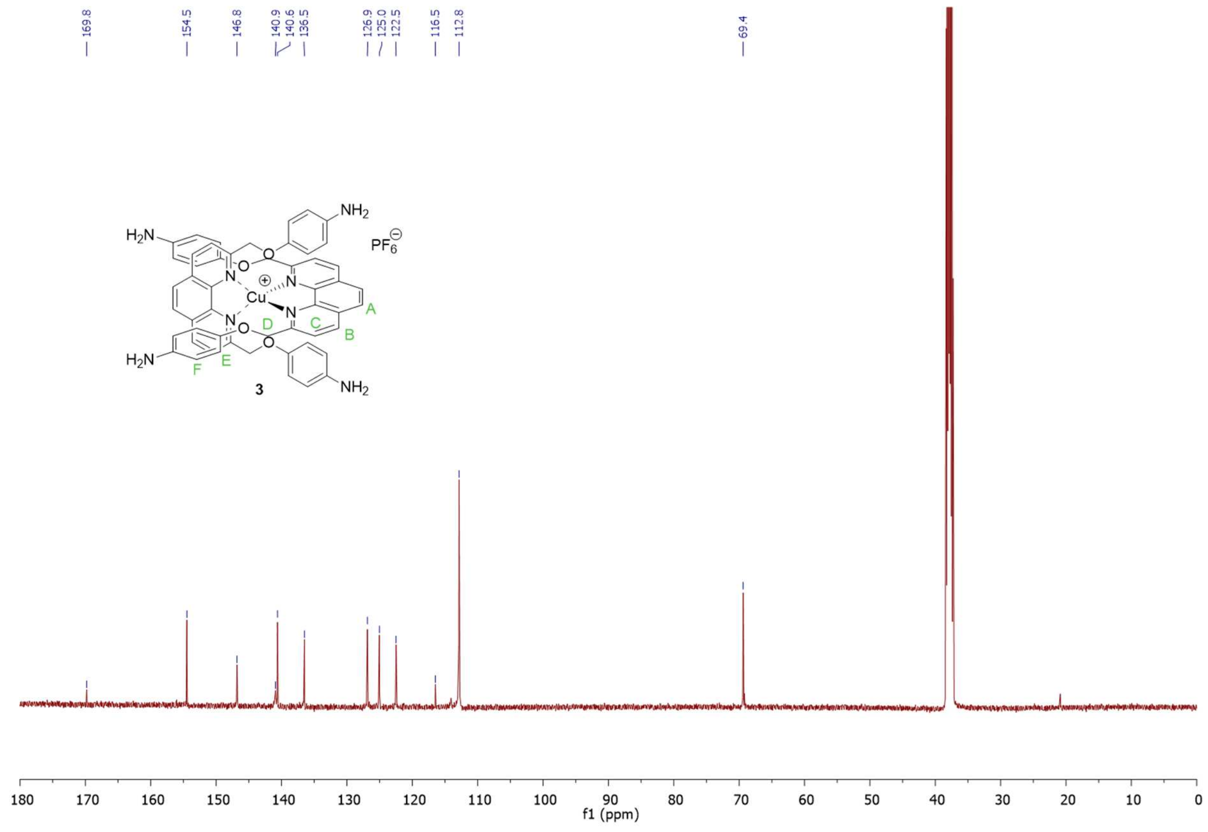

^{13}C NMR spectrum of **3**, 126 MHz, 298 K, DMSO-d_6

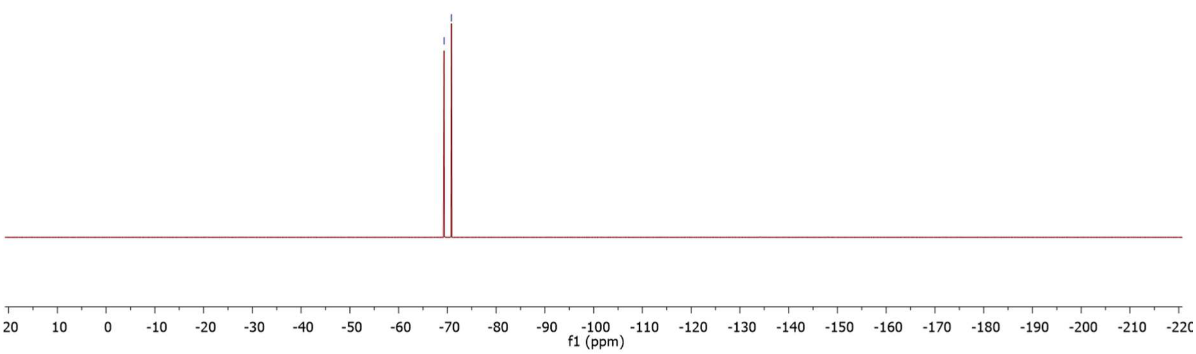

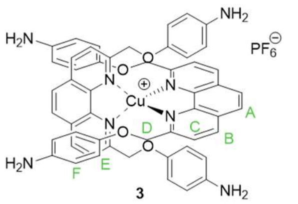

^{19}F NMR spectrum of **3**, 470 MHz, 298 K, DMSO-d_6

Preparation of compound 1a

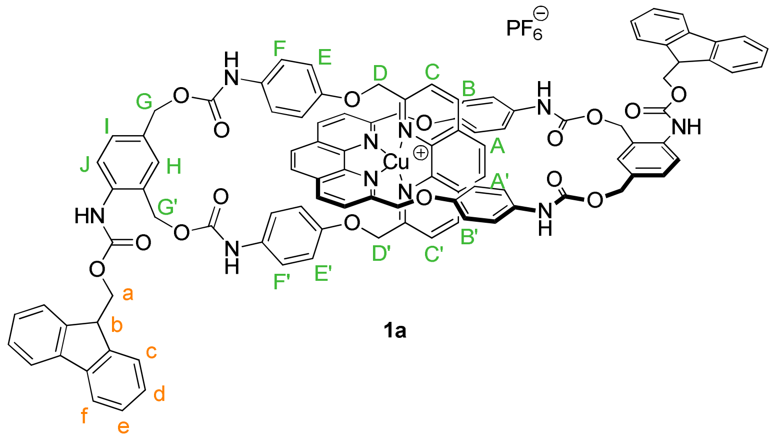

1a

A solution of **4** (100 mg, 237 µmol, 2.2 equiv.), and [Cu(MeCN)$_4$PF$_6$] (40.1 mg, 108 µmol, 1.0 equiv.) in MeCN (20 mL) was stirred for 1 hour. The solution was diluted with DMF (100 mL) and stirred for another hour at 40 °C. Compound **5a** (167 mg, 237 µmol, 2.2 equiv.) and HOBt (32 mg, 237 µmol, 2.2 equiv.) were added with DMF (100 mL). The solution was stirred at 40 °C for 4 days. The solution was concentrated to a minimum of solvent, then added dropwise in Et$_2$O (150 mL). The red precipitate was filtrated, dissolved in DCM, filtrated again and the filtra was concentrated *in vacuo*. The residue was purified by column chromatography over silica gel (DCM/MeOH gradient elution 100:0 to 97:3, R_f = 0.3 (95:5)) to afford catenane **1a** (59.0 mg, 29 %) as a red solid.

^{1}H NMR (500 MHz, DMSO-d_6) δ 9.37-9.26-9.09 (3bs, 6H, H$_{N\mathit{H}COO\ carbamates}$), 8.36 (bs, 4H, H$_{B\text{-}B'}$), 7.98 (bs, 4H, H$_{C\text{-}C'}$), 7.92 (d, 4H, J = 7.5, H$_f$), 7.78 (bs, 6H, H$_{J\text{-}c}$), 7.58 (bs, 4H, H$_{H\text{-}I}$), 7.44 (t, 4H, J = 7.3, H$_e$), 7.35 (t, 4H, J = 7.4, H$_d$), 7.17 (bs, 4H, H$_{A\text{-}A'}$), 6.27 – 6.21 (2d, 8H, J = 8.4, H $_{E\text{-}E'\ or\ F\text{-}F'}$), 5.63 (bs, 8H, H $_{E\text{-}E'\ or\ F\text{-}F'}$), 5.33 (s, 4H, H$_{G\ or\ G'}$), 5.27 (s, 4H, H$_{G\ or\ G'}$), 4.74 (bs, 8H, H$_{D\text{-}D'}$), 4.53 (d, 4H, J = 5.0, H$_a$), 4.38 (m, 2H, H$_b$).

^{13}C NMR (126 MHz, DMSO-d_6) δ 171.61-154.5-153.2-153.0-152.7-143.8-142.4-140.9 (C$_{quat.}$), 138.0 (C$_{B\text{-}B'}$), 136.3, 134.0 (C$_b$), 131.2, 128.2 (C$_e$), 127.8 (C$_d$), 127.2 (C$_{A\text{-}A'\text{-}C\text{-}C'}$), 125.7 (C$_c$), 125.3 (C$_f$), 120.3 (C$_c$), 117.5 (C $_{E\text{-}E'\ or\ F\text{-}F'}$), 111.7 (C $_{E\text{-}E'\ or\ F\text{-}F'}$), 71.5-66.1 (C$_{G\text{-}G'}$), 65.9-65.1 (C$_{D\ or\ D'\text{-}a}$), 61.4 (C$_{D\ or\ D'}$), 46.8 (C$_b$).

^{19}F NMR (376 MHz, DMSO-d_6) δ -70.13 (d, J = 711.4, PF$_6^-$).

HRMS (ESI$^+$) m/z = 1761.4890 [M-PF$_6$]$^+$ (calc. for $C_{102}H_{78}CuN_{10}O_{16}$: 1761.4888 [M-PF$_6$]$^+$).

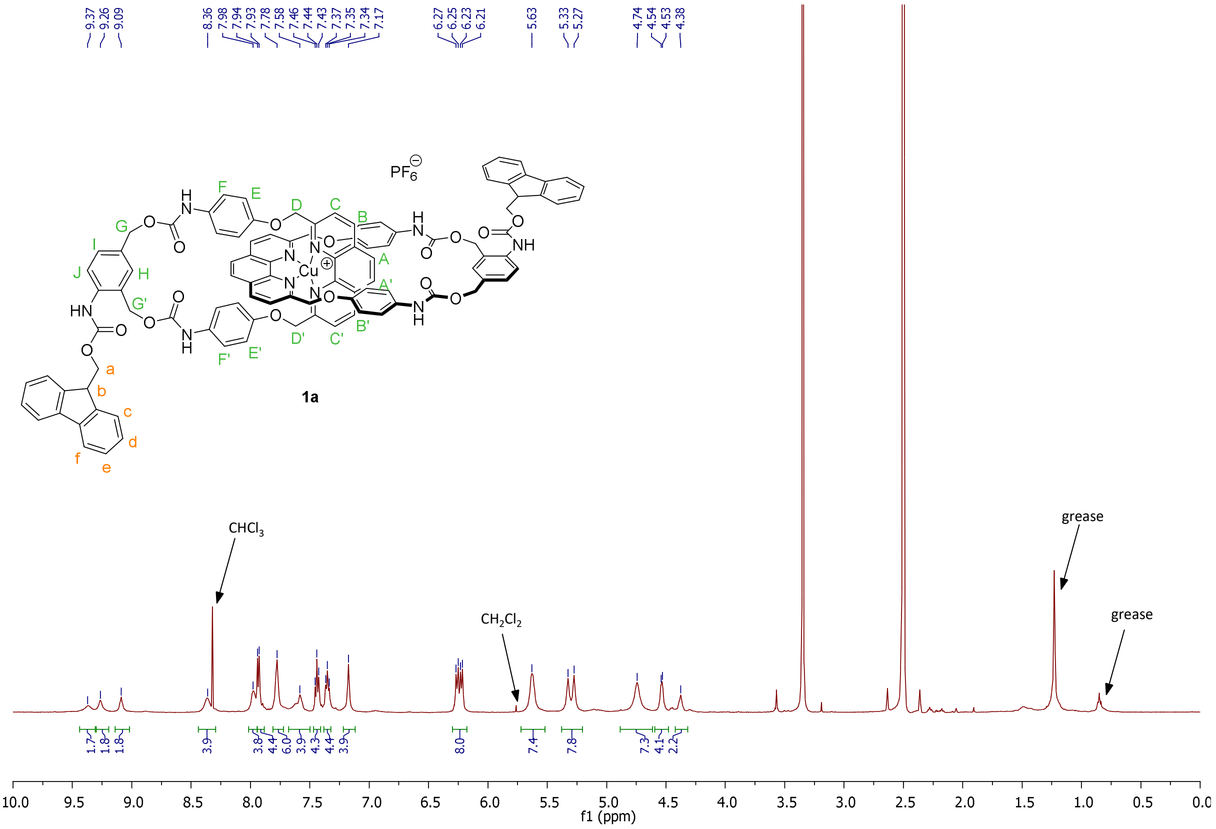

^{1}H NMR spectrum of **1a**, 500 MHz, 298 K, DMSO-*d*6

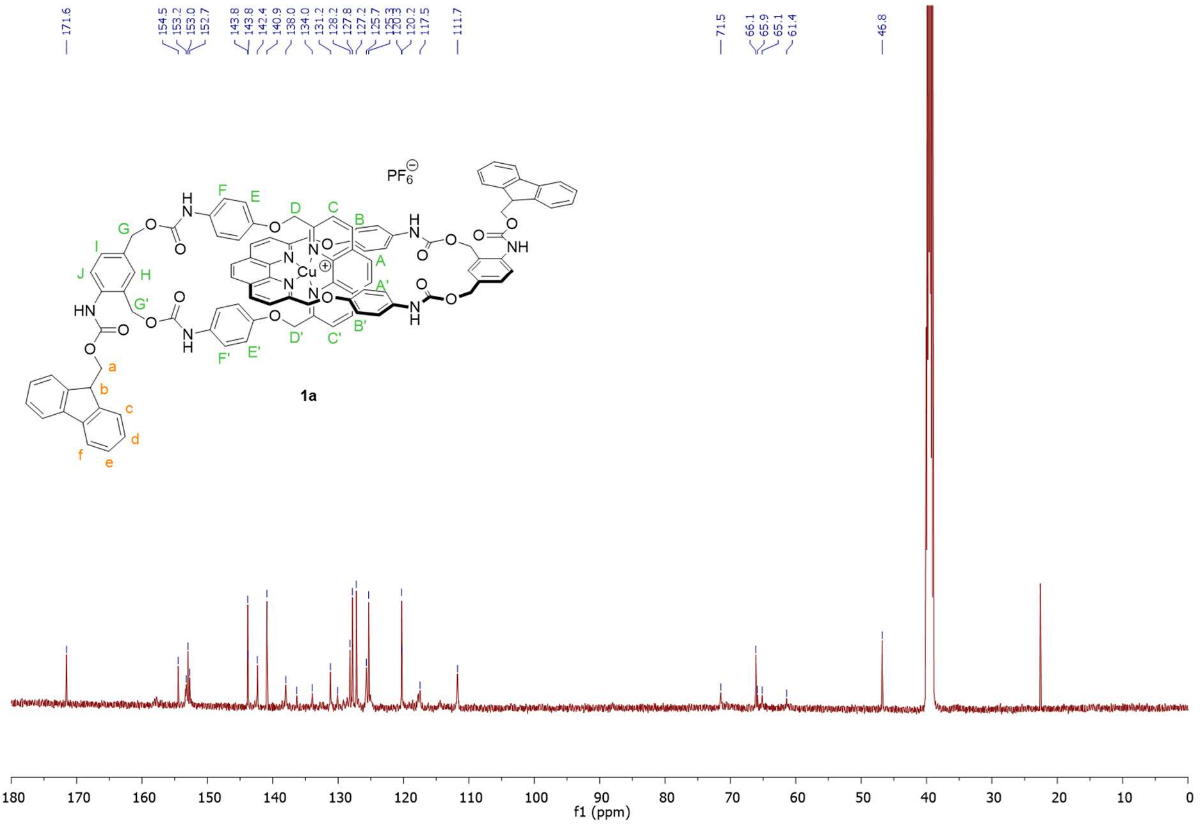

^{13}C NMR spectrum of **1a**, 126 MHz, 298 K, DMSO-*d*6

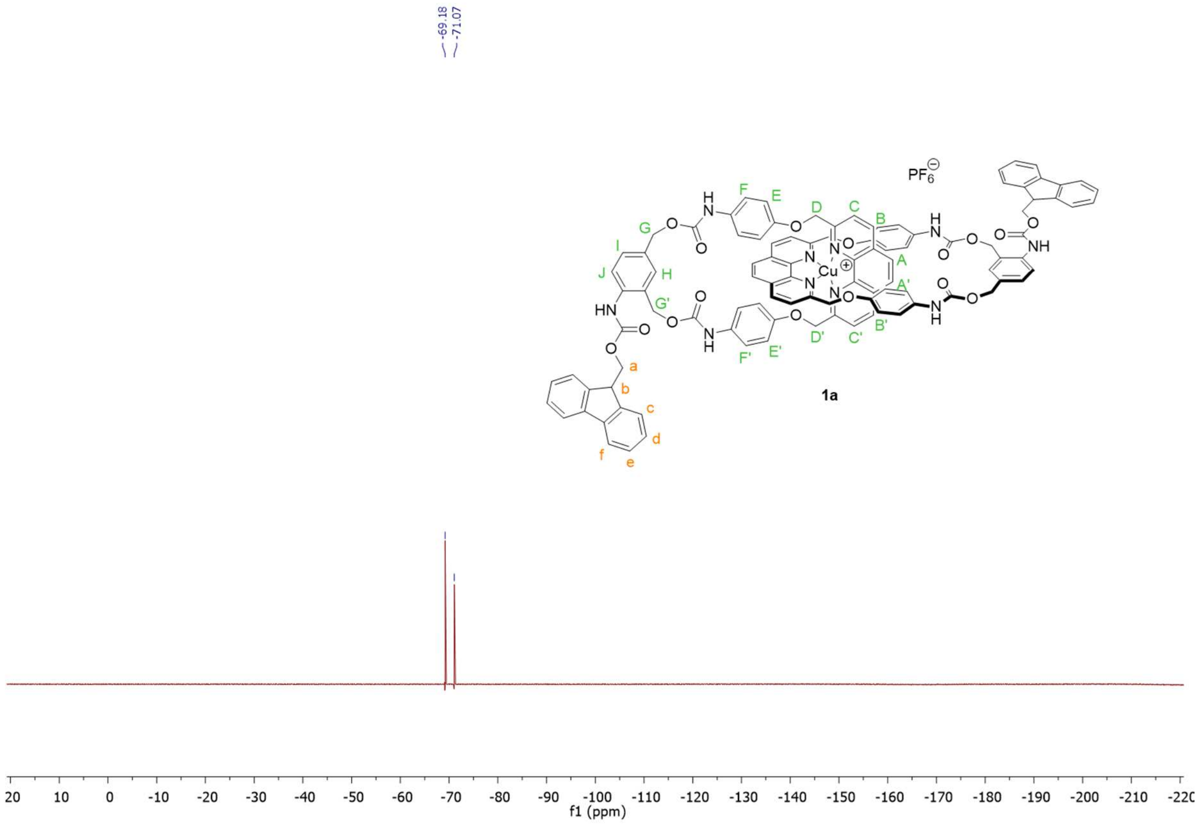

^{19}F NMR spectrum of **1a**, 376 MHz, 298 K, DMSO-d_6

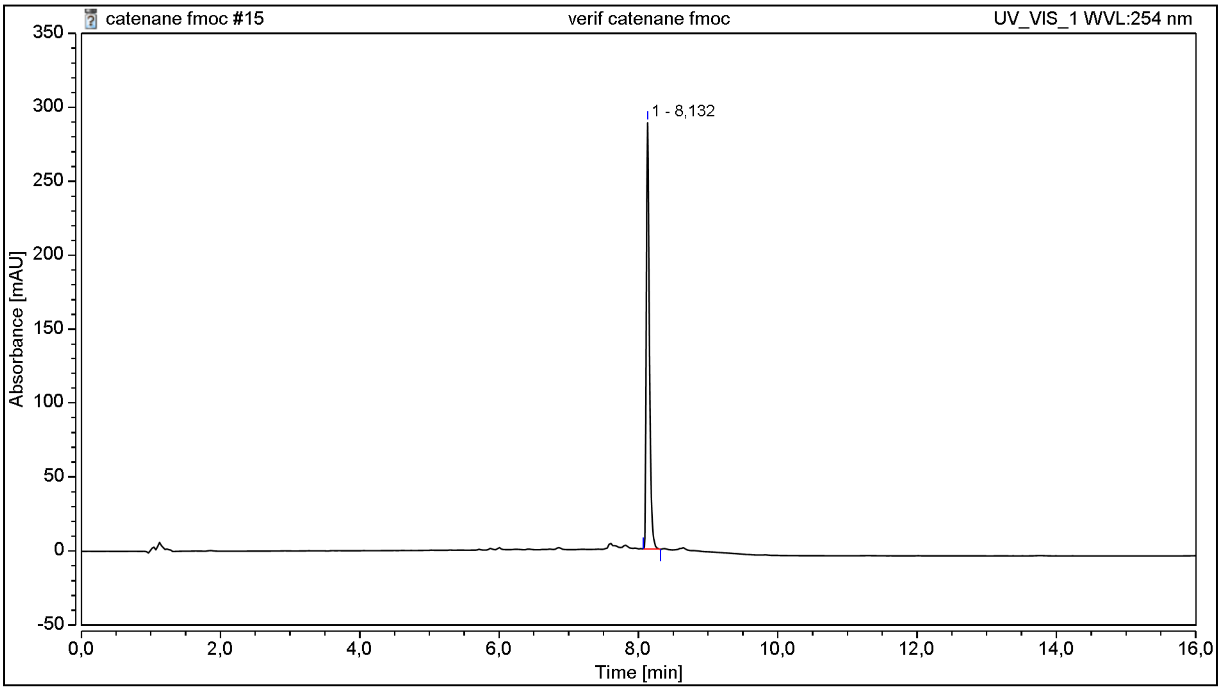

HPLC chromatogram of **1a**

Preparation of compound 1b

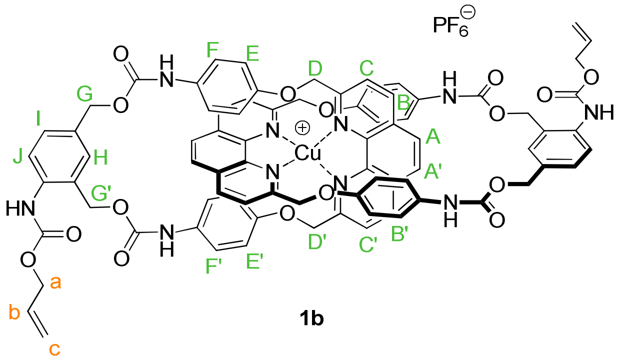

1b

A solution of **4** (280 mg, 663 µmol, 1.1 equiv.), and [Cu(MeCN)$_4$PF$_6$] (112 mg, 301 µmol, 0.5 equiv.) in MeCN (60 mL) was stirred for 1 hour. The solution was diluted with DMF (250 mL) and stirred for another hour at 40 °C. Compound **5b** (376 mg, 663 µmol, 1.1 equiv.) and HOBt (89.6 mg, 663 µmol, 1.1 equiv.) were added with DMF (250 mL). The solution was stirred at 40 °C for 4 days. The solution was concentrated *in vacuo* to a minimum of solvent, then added dropwise in Et$_2$O (300 mL). The red precipitate was filtrated, dissolved in DCM, filtrated again and the filtra was concentrated *in vacuo*. The residue was purified by column chromatography over silica gel (DCM/MeOH gradient elution 100:0 to 97:3, R_f = 0.3 (90:10)) to afford catenane **1b** (223 mg, 45%) as a red solid.

^{1}H NMR (500 MHz, DMSO-d_6) δ 9.24 (bs, 4H, $H_{ArN\mathit{H}CO}$), 9.09 (bs, 2H, $H_{ArN\mathit{H}CO\ alloc}$), 8.37 (bs, 4H, $H_{B\text{-}B'}$), 7.98 (bs, 4H, $H_{C\text{-}C'}$), 7.77 (m, 4H, $H_{H\text{-}I\ or\ J}$), 7.62 (d, 2H, J = 7.2, $H_{I\ or\ J}$), 7.12 (bs, 4H, $H_{A\text{-}A'}$), 6.25-6.21 (2d, 8H, J = 9.0, $H_{E\text{-}E'\ or\ F\text{-}F'}$), 6.03 (bm, 2H, H_b), 5.63 (bs, 8H, $H_{E\text{-}E'\ or\ F\text{-}F'}$), 5.42 (d, 2H, J = 17.1, H_c), 5.34 (s, 4H, $H_{D\ or\ D'}$), 5.28-5.26 (m, 6H, $H_{D\ or\ D'\text{-}c}$), 4.74-4.69 (m, 12H, $H_{G\text{-}G'\text{-}a}$).

^{13}C NMR (126 MHz, DMSO-d_6) δ 154.2-153.2-153.1-153.0-152.7-142.4 ($C_{quat.}$) 138.1 ($C_{B\text{-}B'}$), 136.6-133.7 ($C_{quat.}$), 133.4 (C_b), 131.2, 131.1, 130.2 ($C_{H\ or\ I\ or\ J}$), 129.5 ($C_{H\ or\ I\ or\ J}$), 128.2 ($C_{quat.}$),125.7 ($C_{A\text{-}A'\text{-}C\text{-}C'}$), 124.6 ($C_{H\ or\ I\ or\ J}$), 117.8 ($C_{c\text{-}E\text{-}E'\ or\ F\text{-}F'}$), 117.6 ($C_{c.}$), 111.8 ($C_{c\text{-}E\text{-}E'\ or\ F\text{-}F'}$), 71.5 ($C_{G\text{-}G'}$), 65.2-65.0 ($C_{D\ or\ D'\text{-}a}$), 61.5 ($C_{D\ or\ D'}$).

^{19}F NMR (470 MHz, DMSO-d_6) δ -70.09 (d, J = 711.0, PF_6^-).

HRMS (ESI$^+$) *m/z* = 1485.3944 [M-PF$_6$]$^+$ (calc. for $C_{80}H_{66}CuN_{10}O_{16}$: 1485.3949 [M-PF$_6$]$^+$).

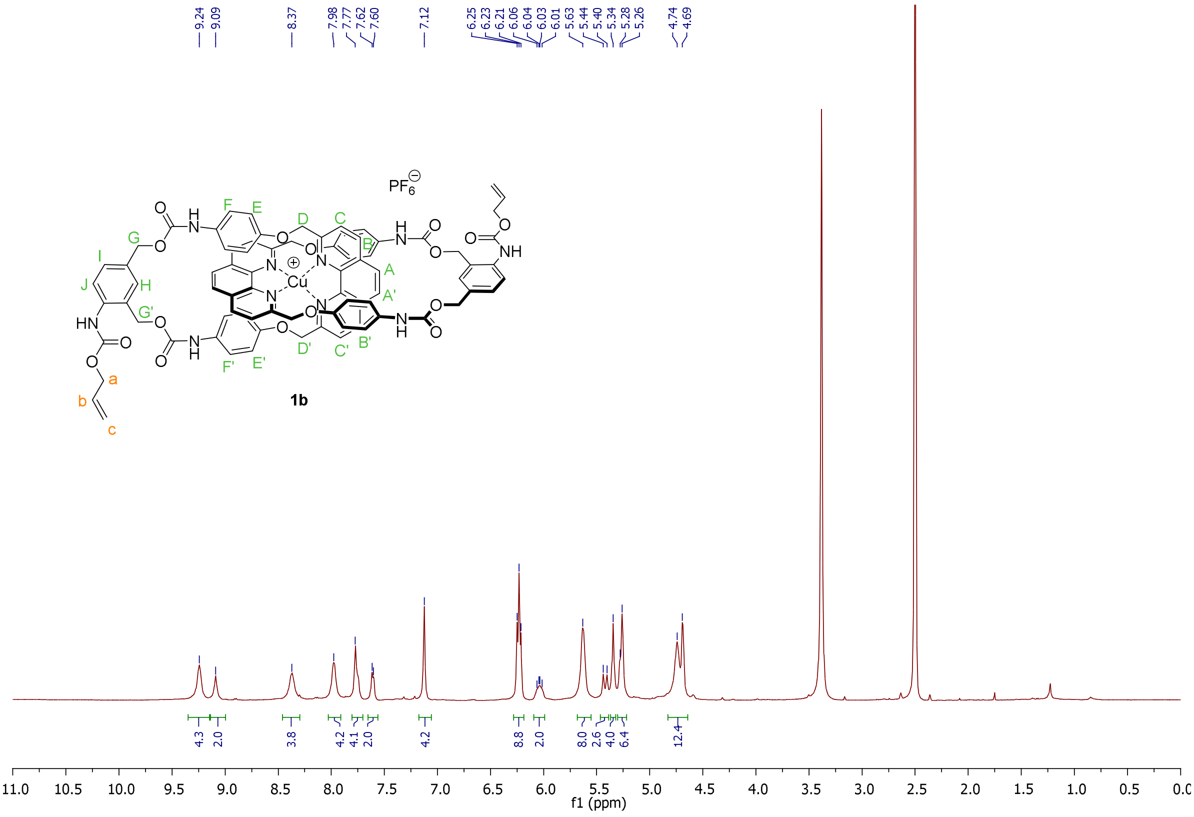

^{1}H NMR spectrum of **1b**, 500 MHz, 298 K, DMSO-d_6

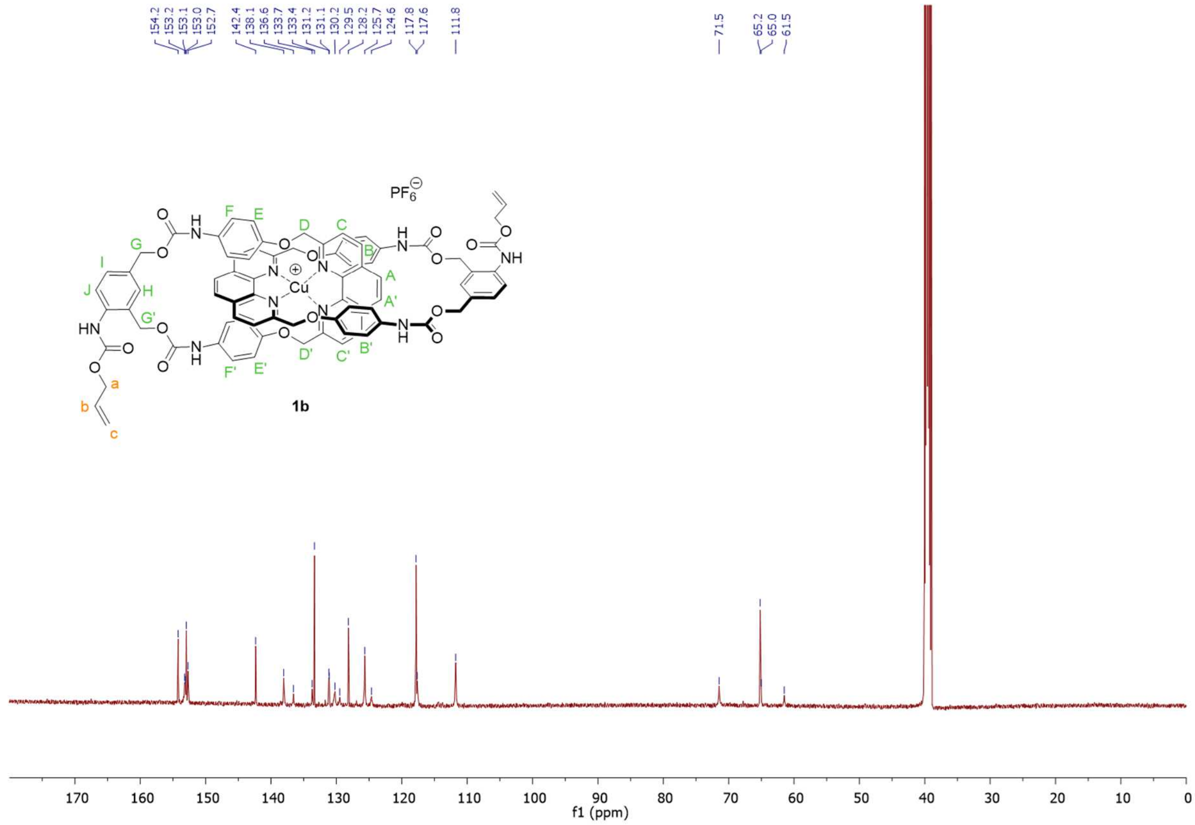

^{13}C NMR spectrum of **1b**, 126 MHz, 298 K, DMSO-d_6

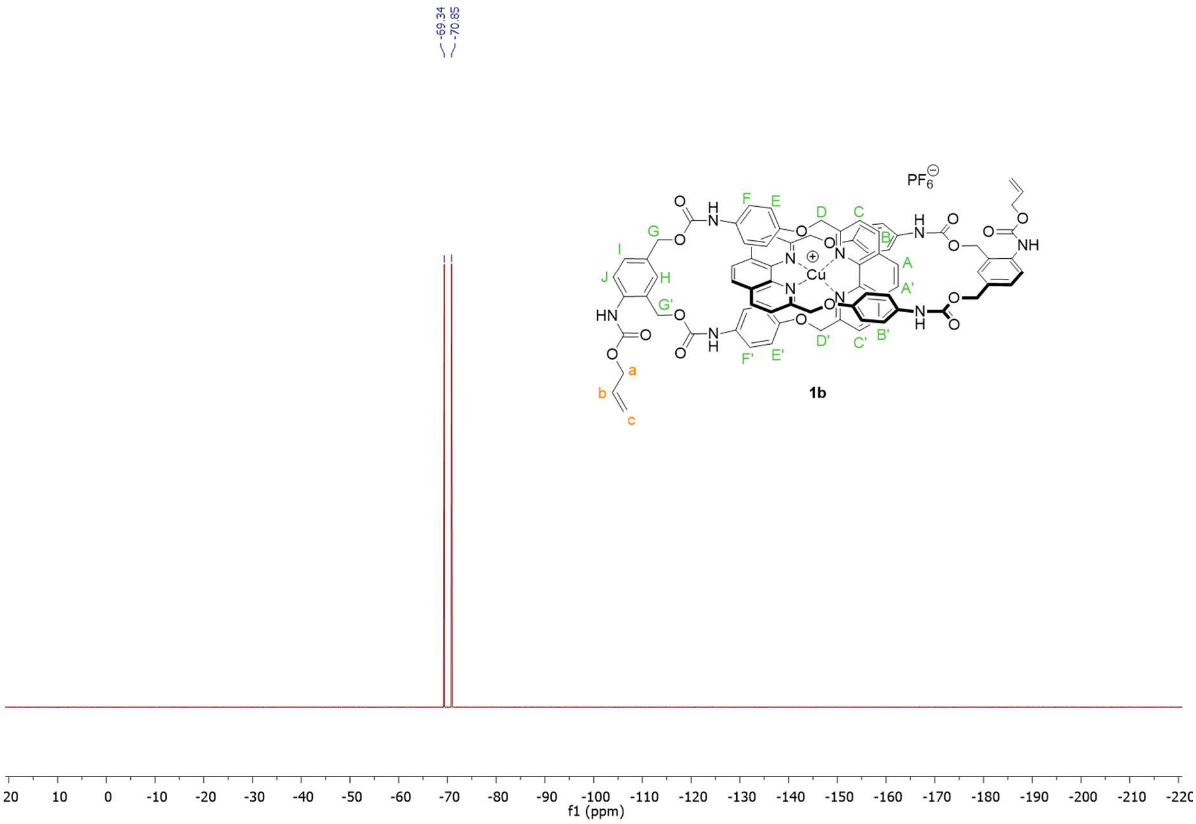

^{19}F NMR spectrum of **1b**, 470 MHz, 298 K, DMSO-*d*$_6$

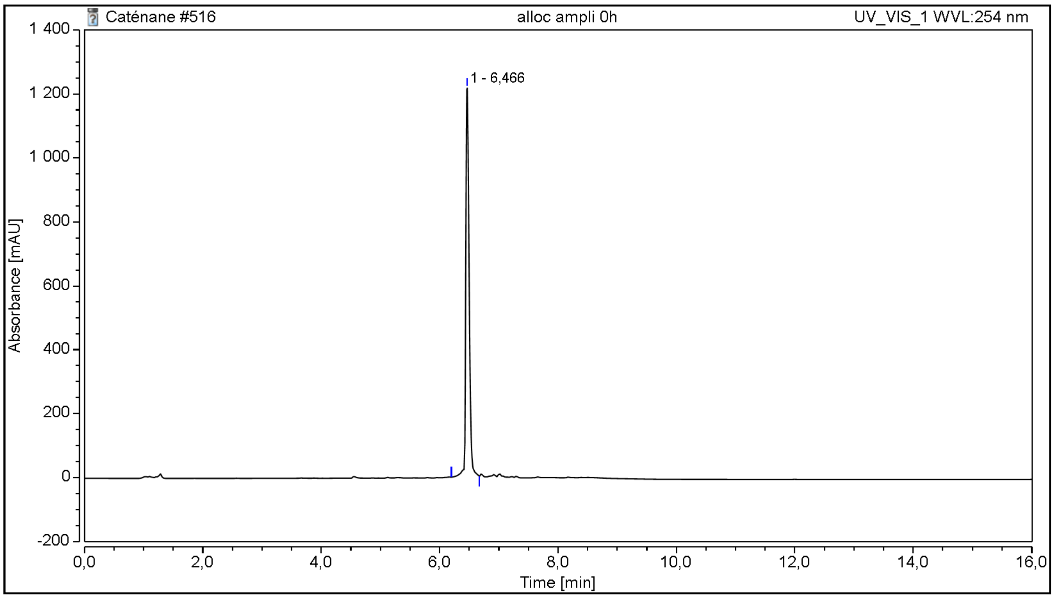

HPLC chromatogram of **1b**

Preparation of compound 10

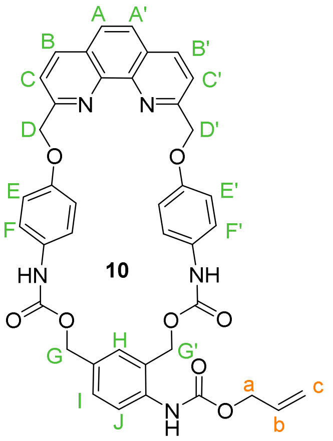

To a solution of **4** (50.0 mg, 118 µmol, 1.6 equiv.) in DMF (10 mL) was added compound **5b**[1] (42.0 mg, 74 µmol, 1.0 equiv.) and HOBt (10.0 mg, 74 µmol, 1.0 equiv.). Solution was stirred at 30 °C for 8 hours then DMF (50 mL) was added. Solution was stirred for 4 days then solvent was evaporated. The crude was diluted in DCM (10 mL) and organic layer was washed with a saturated $NaHCO_{3(aq)}$ solution (3 x 5 mL), dried over $MgSO_4$ and concentrated *in vacuo*. Purification by column chromatography over silica gel (DCM/MeOH gradient elution 100:0 to 95:5, R_f = 0.3 (95:5)) afforded compound **10** (19.0 mg, 36%) as a white solid.

^{1}H NMR (500 MHz, DMSO-d_6) δ 9.59-9.50-9.20 (3bs, 3H, $H_{NHCOO\ carbamates}$), 8.48 (2d, 2H, J = 8.3, $H_{B-B'}$), 7.96 (s, 2H, $H_{A-A'}$), 7.88 (2d, 2H, J = 8.3, $H_{C-C'}$), 7.34 – 7.15 (m, 11H, $H_{E-E'-F-F'-H-I-J}$), 5.96 (ddt, 1H, J_{trans} = 17.4, J_{cis} = 10.4, J = 5.4, H_b), 5.55 (s, 4H, $H_{D-D'}$), 5.35 (d, 1H, J_{trans} = 17.4, H_c), 5.22 (d, 1H, J_{cis} = 10.4, H_c), 5.11-5.07 (2s, 4H, $H_{G-G'}$), 4.57 (d, 2H, J = 5.4, H_a). *(Even after carrefull evaporation, pics of residual DMF and DCM were present in the spectrum)*

^{13}C NMR (126 MHz, DMSO-d_6) δ 162.4-157.4-154.2-153.9-153.5-144.4 ($C_{quat.}$), 137.3 ($C_{B-B'}$), 134.4-134.1 ($C_{quat.}$), 133.3 (C_b), 132.3-132.2 ($C_{quat.}$), 127.9 ($C_{quat.}$), 127.0, 126.6 ($C_{A-A'}$), 125.6, 124.5, 122.6 ($C_{C-C'}$), 120.5, 120.0, 117.6 (C_c), 115.7, 71.7 ($C_{D-D'}$), 65.0 (C_a), 64.4-60.9 ($C_{G-G'}$). *(Even after carrefull evaporation, pics of residual DMF and DCM were present in the spectrum)*

HRMS (ESI$^+$) m/z = 712.2398 $[M+H]^+$ (calc. for $C_{40}H_{34}N_5O_8$: 712.2402 $[M+H]^+$).

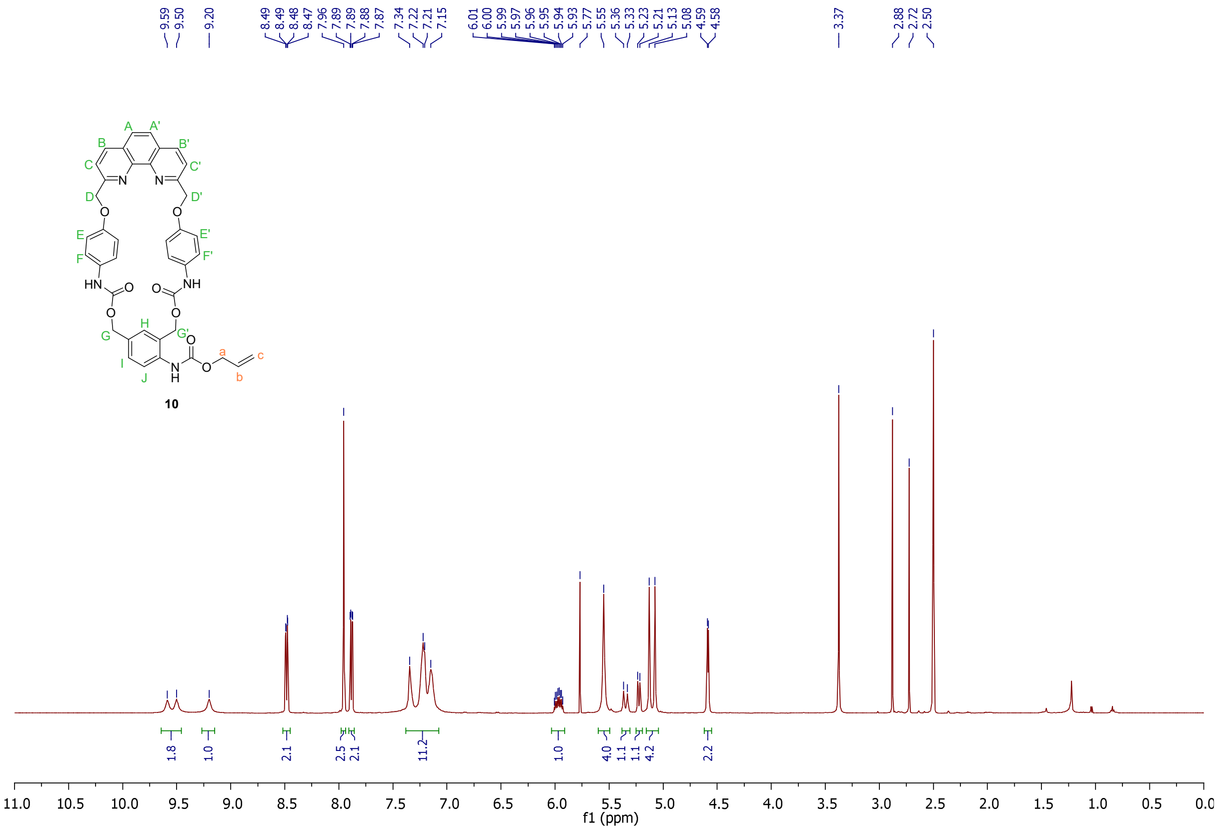

^{1}H NMR spectrum of **10**, 500 MHz, 298 K, DMSO-*d*$_6$

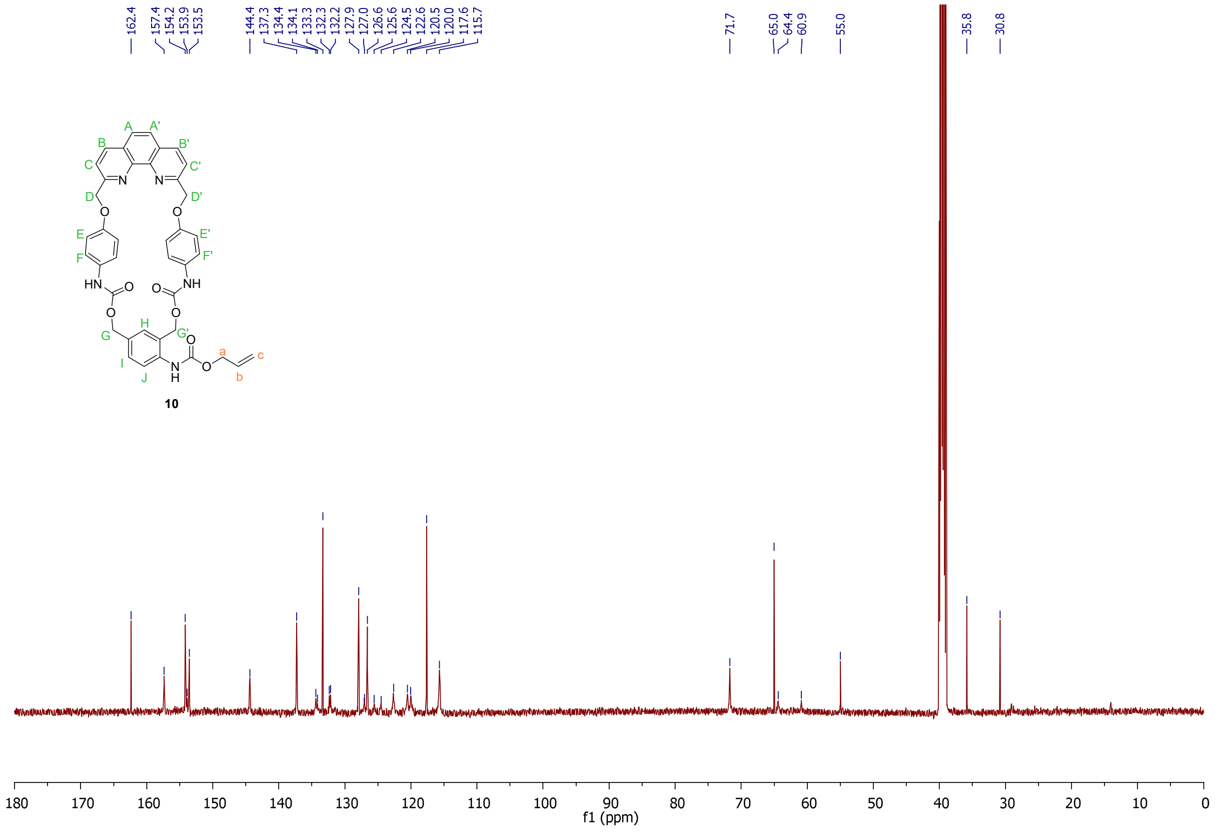

^{13}C NMR spectrum of **10**, 126 MHz, 298 K, DMSO-*d*$_6$

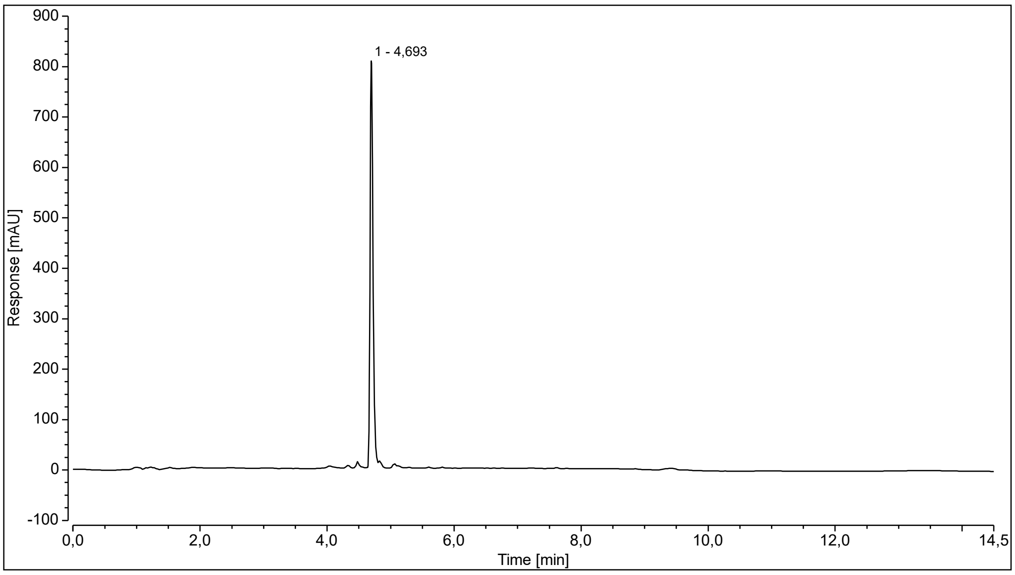

HPLC chromatogram of **10**

Preparation of compound 11

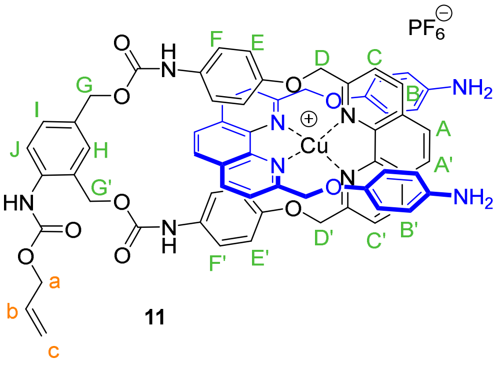

11

To a solution of marocycle **10** (29.0 mg, 40 µmol, 1.0 equiv.) in MeCN (4 mL) was added $[Cu(MeCN)_4]PF_6$ (15.0 mg, 40 µmol, 1.0 equiv.). The solution was stirred for 30 minutes at room temperature and phenantroline **4** (17.2 mg, 40 µmol, 1 equiv) was added. The resulting dark red solution was stirred for two hours at room temperature and the solvent was evaporated. Purification by column chromatography over silica gel (DCM/MeOH gradient elution 100:0 to 95:5, R_f = 0.3 (95:5)) afforded **11** (30.0 mg, 56%) as a dark red solid.

^{1}H NMR (500 MHz, DMSO-*d*6) δ 9.29-9.23-9.07 (3bs, 3H $H_{NHCOO\ carbamates}$), 8.91 (d, 2H, *J* = 8.1, $H_{B-B'}$), 8.32 (s, 4H, $H_{A-A'-B-B'}$), 8.26 (d, 2H, *J* = 8.1, $H_{C-C'}$), 7.90 (d, 2H, *J* = 7.2, $H_{C-C'}$), 7.73 – 7.61 (2m, 3H, H_{H-I-J}), 7.09 (s, 2H, $H_{A-A'}$), 6.20 (m, 4H, $H_{E-E'}$),6.14 (d, 4H, *J* = 8.8, $H_{E-E'}$), 6.04 (d, 5H, J = 8.8, $H_{F-F'}$, H_b); 5.49 (m, 4H, $H_{F-F'}$), 5.42 (d, 1H, J_{trans} = 17.3, H_c),5.35 (m, 2H, $H_{D\ or\ D'}$), 5.27 (m, 3H, H_c, $H_{D\ or\ D'}$),4.84 (m, 4H, $H_{G-G'}$), 4.70, (m, 6H, H_a,$H_{D-D'}$), 4.58 (bs, 4H, H_{NH2}).

^{13}C NMR (126 MHz, DMSO-*d*6) δ 154.9-154.2-153.1-152.6-148.5143.0-142.9-141.6 ($C_{quat.}$), 138.5 ($C_{B-B'}$), 138.0 ($C_{B-B'}$), 133.6 ($C_{quat.}$), 133.3 (C_b), 131.3 ($C_{quat.}$), 130.3 ($C_{H\ or\ I\ or\ J}$), 128.9 ($C_{H\ or\ I\ or\ J}$), 128.0 ($C_{quat.}$), 127.1 ($C_{A-A'}$), 126.6 ($C_{C-C'}$), 125.4 ($C_{A-A'}$), 124.6 ($C_{H\ or\ I\ or\ J}$), 122.9 ($C_{C-C'}$), 117.8 (C_c), 117.6 ($C_{E-E'}$), 115.0 ($C_{F-F'}$), 114.5 ($C_{E-E'}$), 111.3 ($C_{F-F'}$), 71.2 ($C_{G-G'}$), 71.1 ($C_{D-D'}$), 65.2 (C_a), 65.1 ($C_{D\ or\ D'}$), 61.3 ($C_{D\ or\ D'}$).

HRMS (ESI$^+$) *m/z* = 1196.3379 $[M-PF_6]^+$ (calc. for $C_{66}H_{55}CuN_9O_{10}$: 1196.3362 $[M-PF_6]^+$).

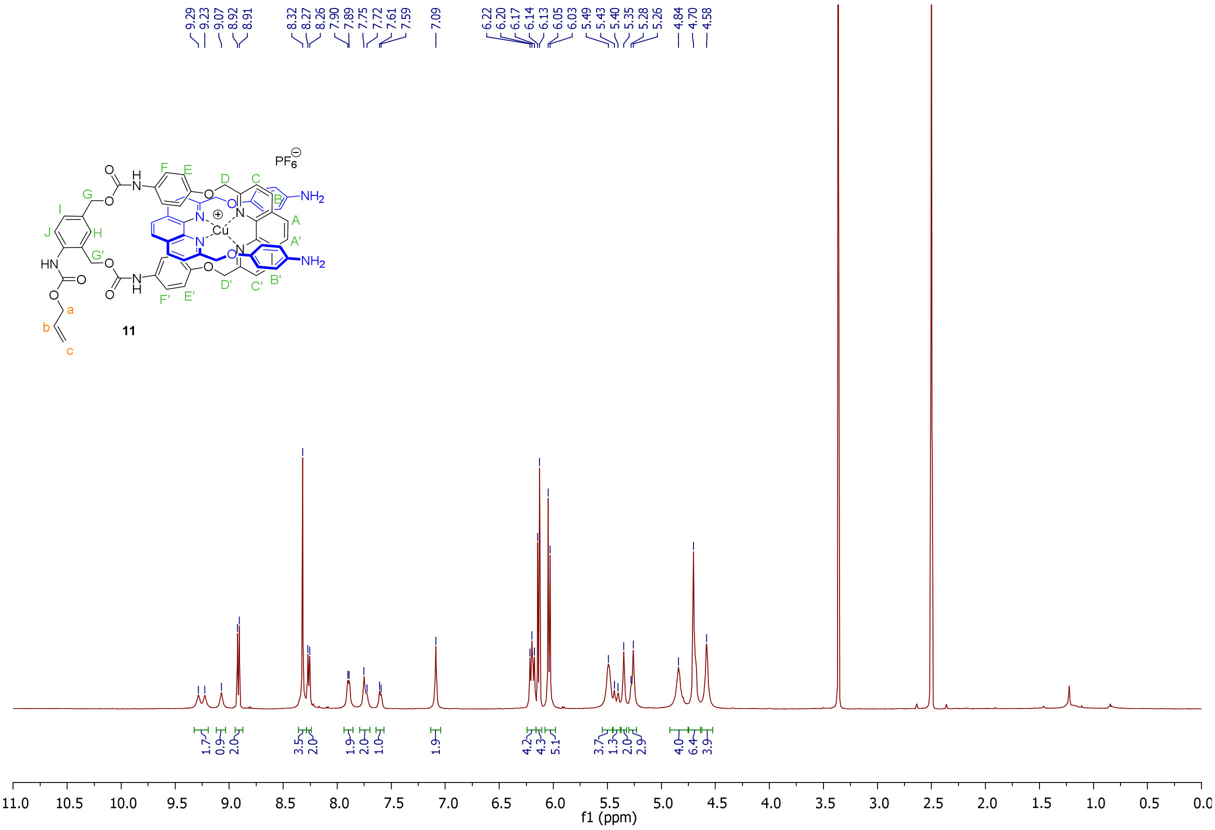

^{1}H NMR spectrum of **11**, 500 MHz, 298 K, DMSO-*d*$_6$

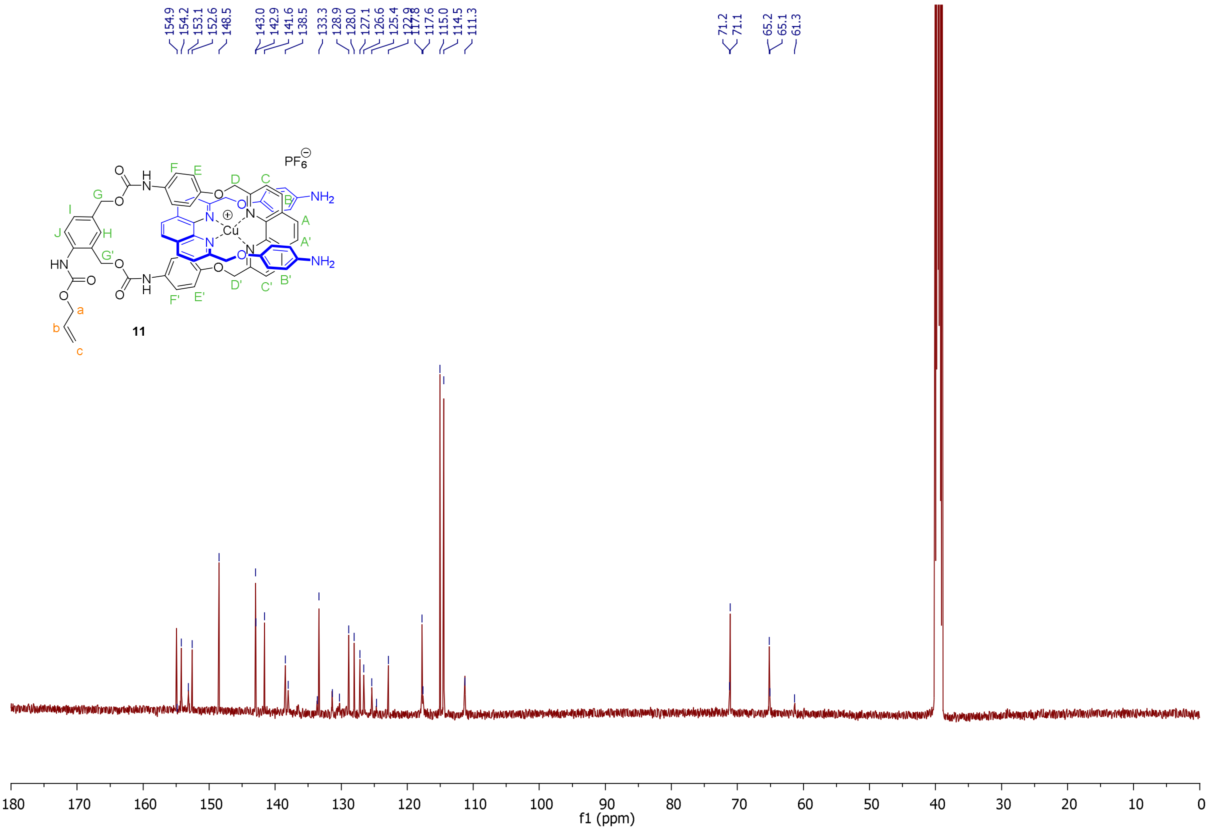

^{13}C NMR spectrum of **11**, 126 MHz, 298 K, DMSO-*d*$_6$

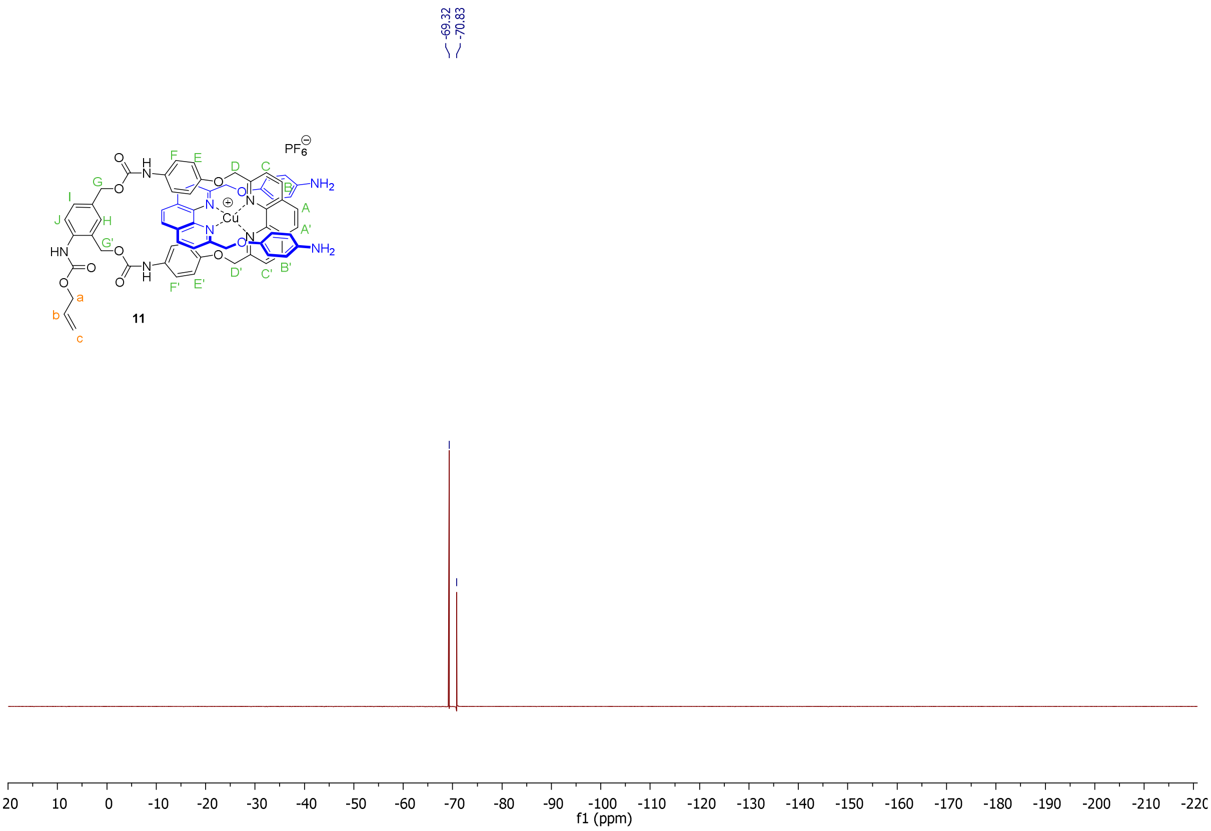

^{19}F NMR spectrum of **11**, 470 MHz, 298 K, DMSO-d_6

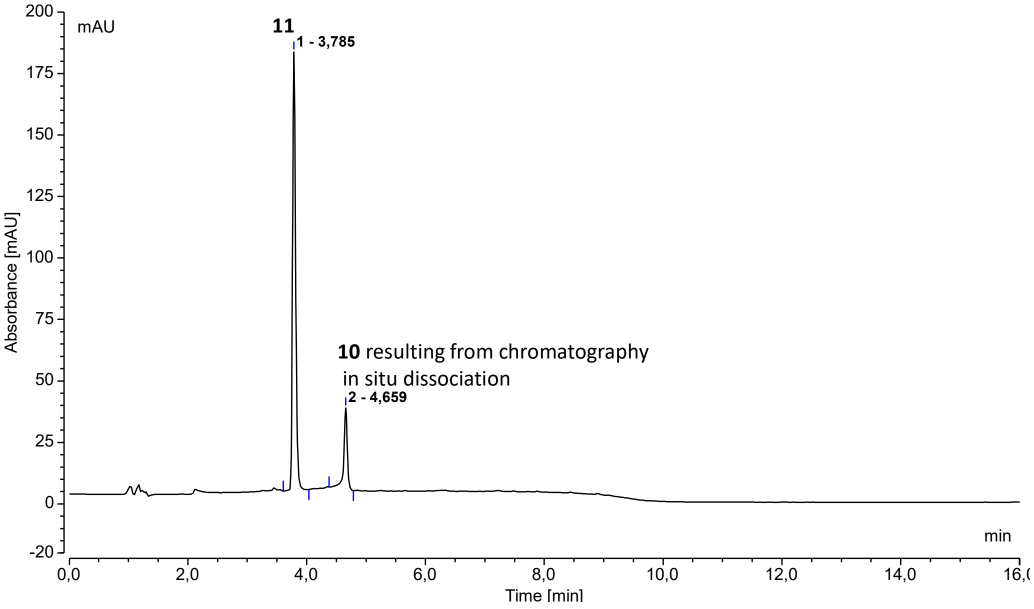

HPLC chromatogram of **11**

II. Study of catenanes 1a and 1b

II.1. Stability of catenanes 1a and 1b

A solution of catenane **1a** (5 mg, 2.62 µmol, 1.0 equiv.) in dry EtOH/DMSO 8:2 (262 µL) was heated to 60 °C and the stability was monitored by HPLC. After 48 hours, HPLC showed no decomposition of catenane **1a**.

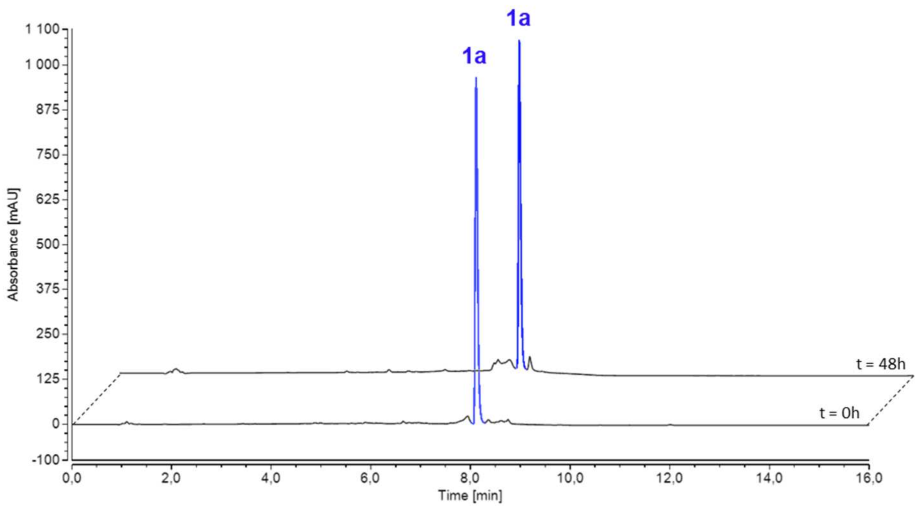

Figure S1: Stacked HPLC of catenane **1a** after 48 hours (rt **1a**: 8.1 min).

A solution of catenane **1b** (5 mg, 3.06 µmol, 1.0 equiv.) in dry EtOH/DMSO/DCE 7:2:1 (262 µL) was heated to 60 °C and the stability was monitored by HPLC. After 48 hours, HPLC showed no decomposition of catenane **1b**.

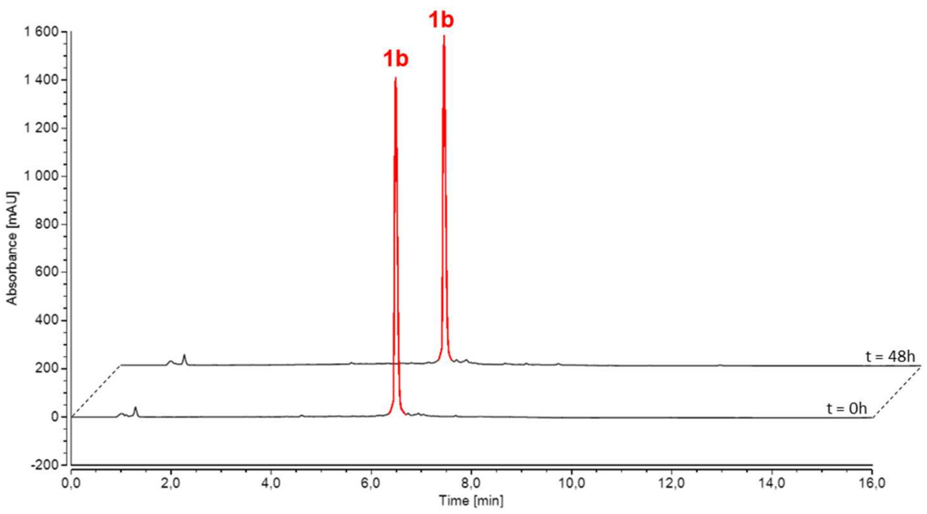

Figure S2: Stacked HPLC of catenane **1b** after 48 hours. Retention time **1b**: 6.5 min.

II.2. HPLC and UPLC-HRMS decomposition of catenanes 1a and 1b

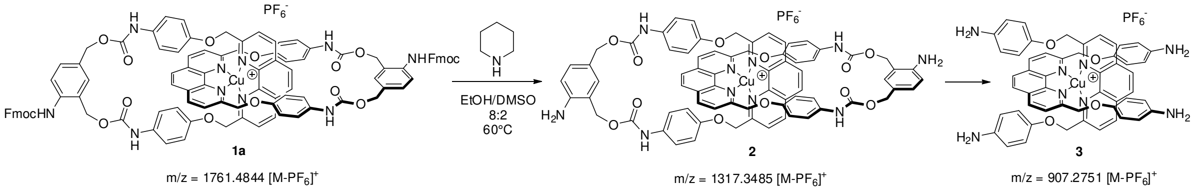

To a solution of catenane **1a** (3 mg, 1.57 µmol, 1.0 equiv.) in dry EtOH/DMSO 8:2 (157 µL) was added piperidine (0.8 µL, 7.86 µmol, 5.0 equiv.). The mixture was heated to 60 °C and the reaction was monitored by HPLC and UPLC-HRMS.

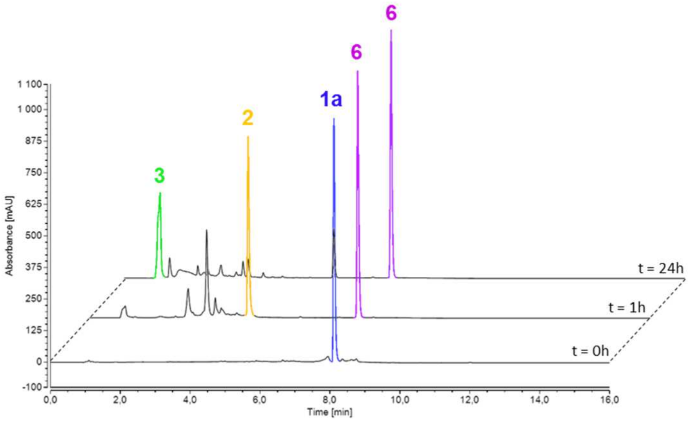

Figure S3: HPLC traces of catenane **1a** over time. Retention times (min) **1a**: 8.1, **2**: 4.5, **3**: 1.0, **6**: 7.6.

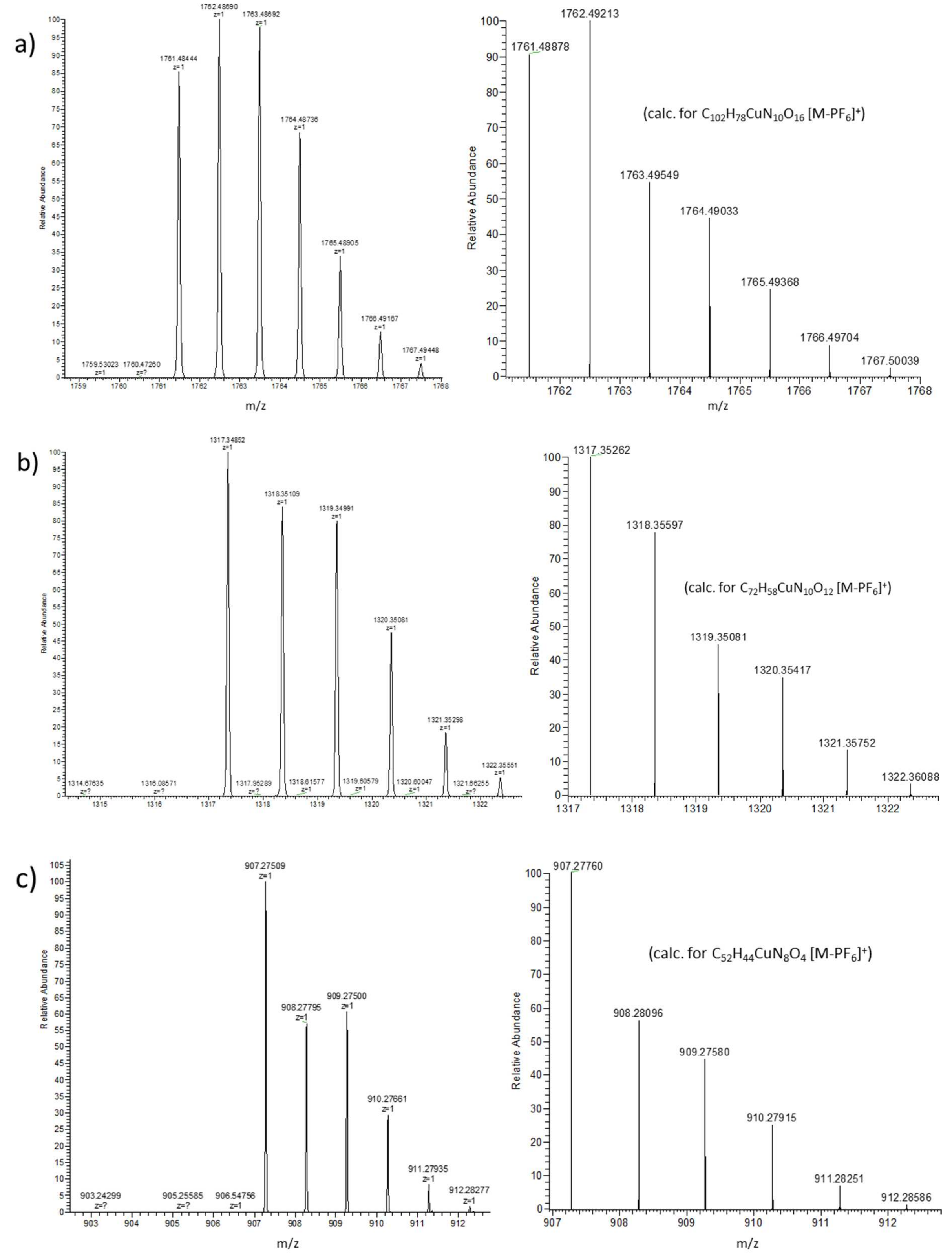

Figure S4: HRMS analysis obtained for the decomposition of catenane **1a** corresponding to compound (a) **1a**; (b) **2**; (c) **3**.

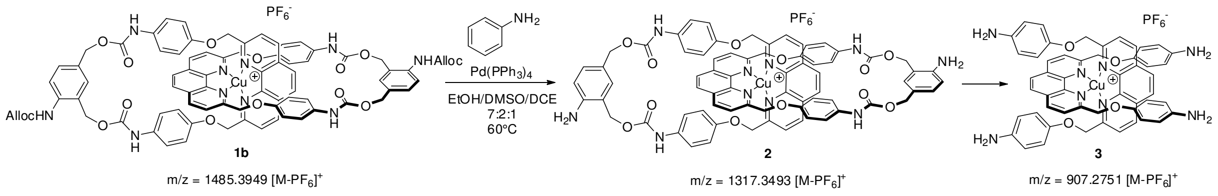

To a solution of catenane **1b** (3 mg, 1.84 µmol, 1.0 equiv.) and aniline (0.84 µL, 9.19 µmol, 5 equiv.) in dry EtOH/DMSO 8:2 (165.6 µL) was added a $9.95.10^{-4}$ $mol.L^{-1}$ solution of $Pd(PPh_3)_4$ in dry DCE (18.4 µL, 18.4 nmol, 0.01 equiv.). The mixture was heated to 60 °C and the reaction was monitored by HPLC and UPLC-HRMS.

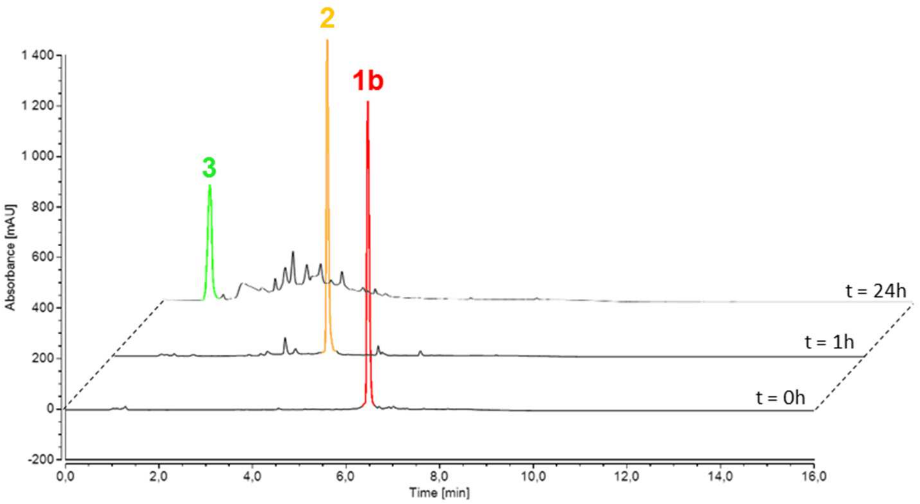

Figure S5: HPLC traces of catenane **1b** over time. Retention times (min) **1b**: 6.5, **2**: 4.5, **3**: 1.0.

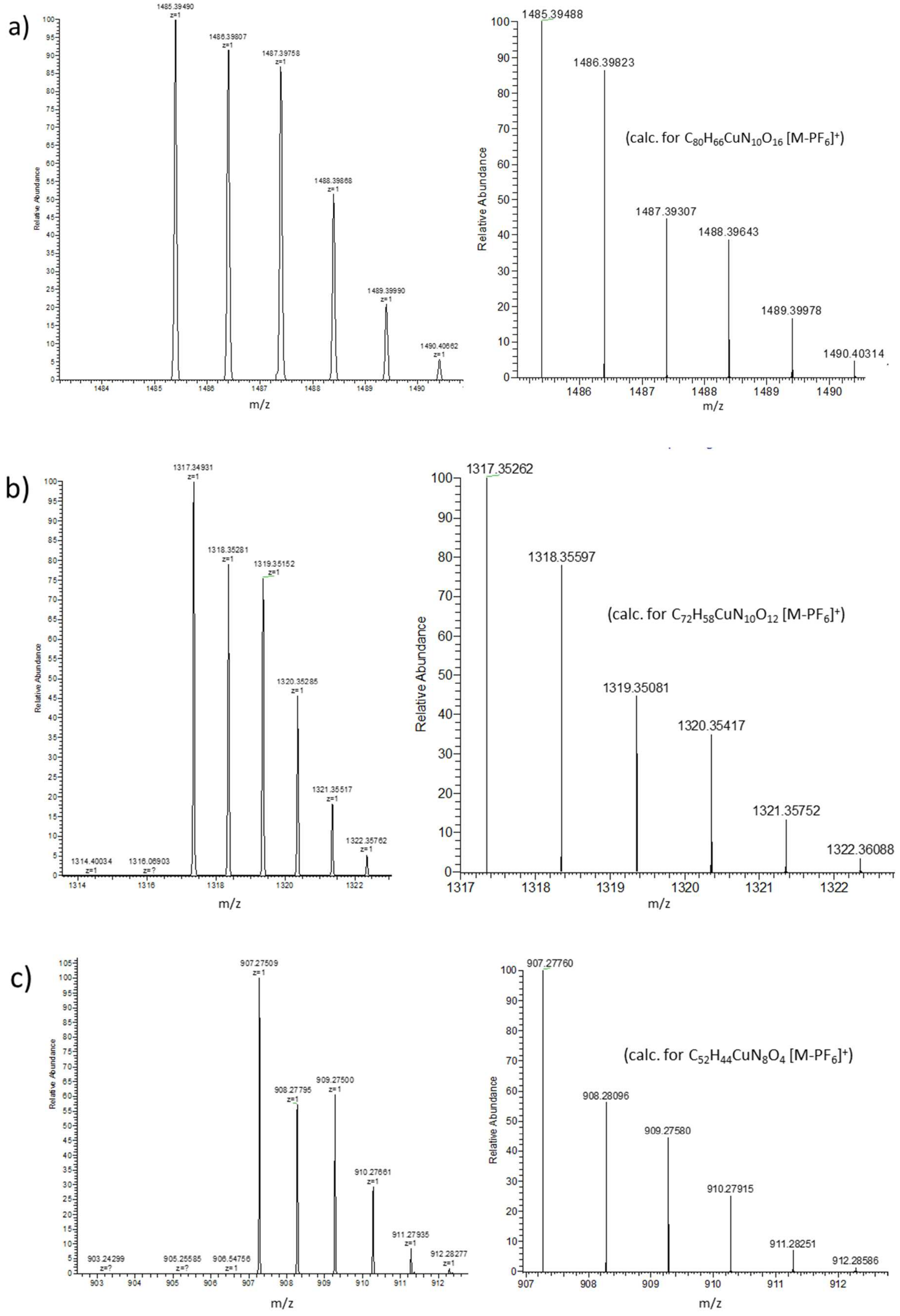

Figure S6: HRMS analysis obtained for the decomposition of catenane **1b** corresponding to compound (a) **1b**; (b) **2**; (c) **3**

II.3. Catalytic activity of compound 3

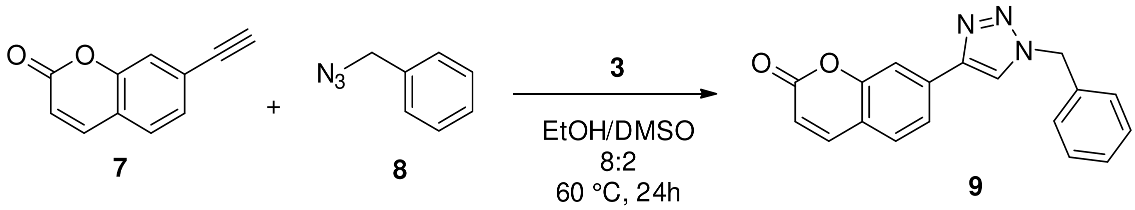

To a solution of compound **3** (10 mg, 9.37 µmol, 1 equiv.) in EtOH/DMSO 8:2 (937 µL) were added coumarine **7** (1.6 mg, 9.37 µmol, 1 equiv.) and azide **8** (1.4 µL, 11.2 µmol, 1.2 equiv.). The mixture was heated to 60 °C and the reaction was monitored with HPLC. After 24 hours, a complete formation of compound **9** was observed.

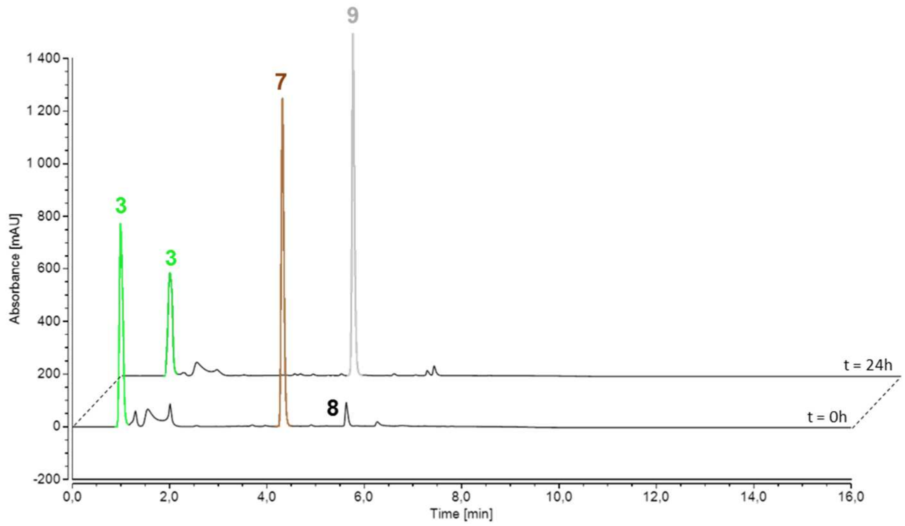

Figure S7: Stacked HPLC of the formation of compound **9** with **3** as catalyst. Retention times (min) **3**: 1.0, **7**: 4.3, **8**: 5.6, **9**: 4.7.

II.4. Catalytic activity of compound 11

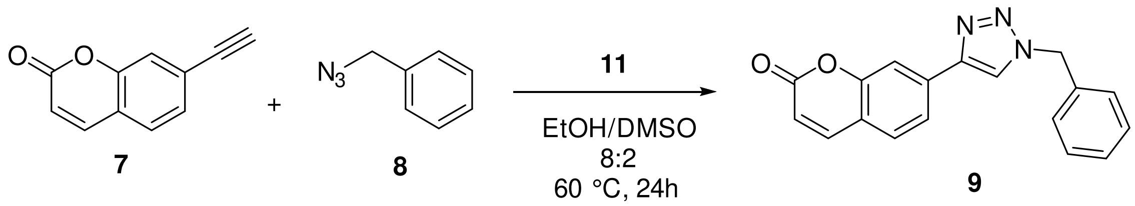

To a solution of **11** (5 mg, 3.72 µmol, 1 equiv.) in EtOH/DMSO 8:2 (372 µL) were added coumarine **7** (6.3 mg, 37.2 µmol, 10 equiv.) and azide **8** (5.6 µL, 44.6 µmol, 12 equiv.). The mixture was heated to 60 °C and the reaction was monitored with HPLC. After 24 hours, only slight formation of compound **9** was observed.

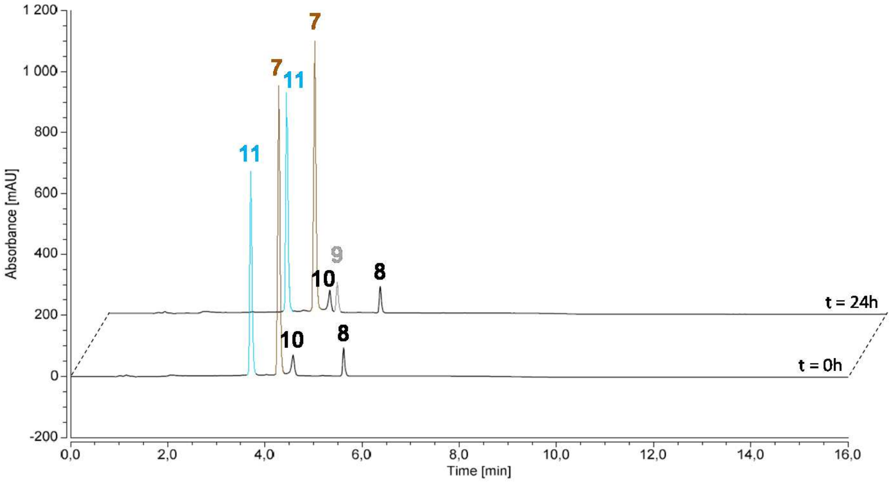

Figure S8: Stacked HPLC of the formation of compound **9** with **11** as catalyst. Retention times (min) **11**: 3.78, **7**: 4.3, **9**: 4.7, **8**: 5.6, **9**: 4.7.

II.5. Activation of catenane 1a

To a solution of catenane **1a** (5 mg, 2.62 µmol, 1 equiv.), coumarine **7** (4.5 mg, 26.2 µmol, 10 equiv.) and azide **8** (3.9 µL, 31.4 µmol, 12 equiv.) in EtOH/DMSO 8:2 (262 µL) was added a 0.1 M solution of piperidine in EtOH/DMSO 8:2 (2.62 µL, 0.26 µmol, 0.1 equiv.). The mixture was heated to 60°C. The same reaction was realized without using piperidine. Both reactions were monitored with HPLC.

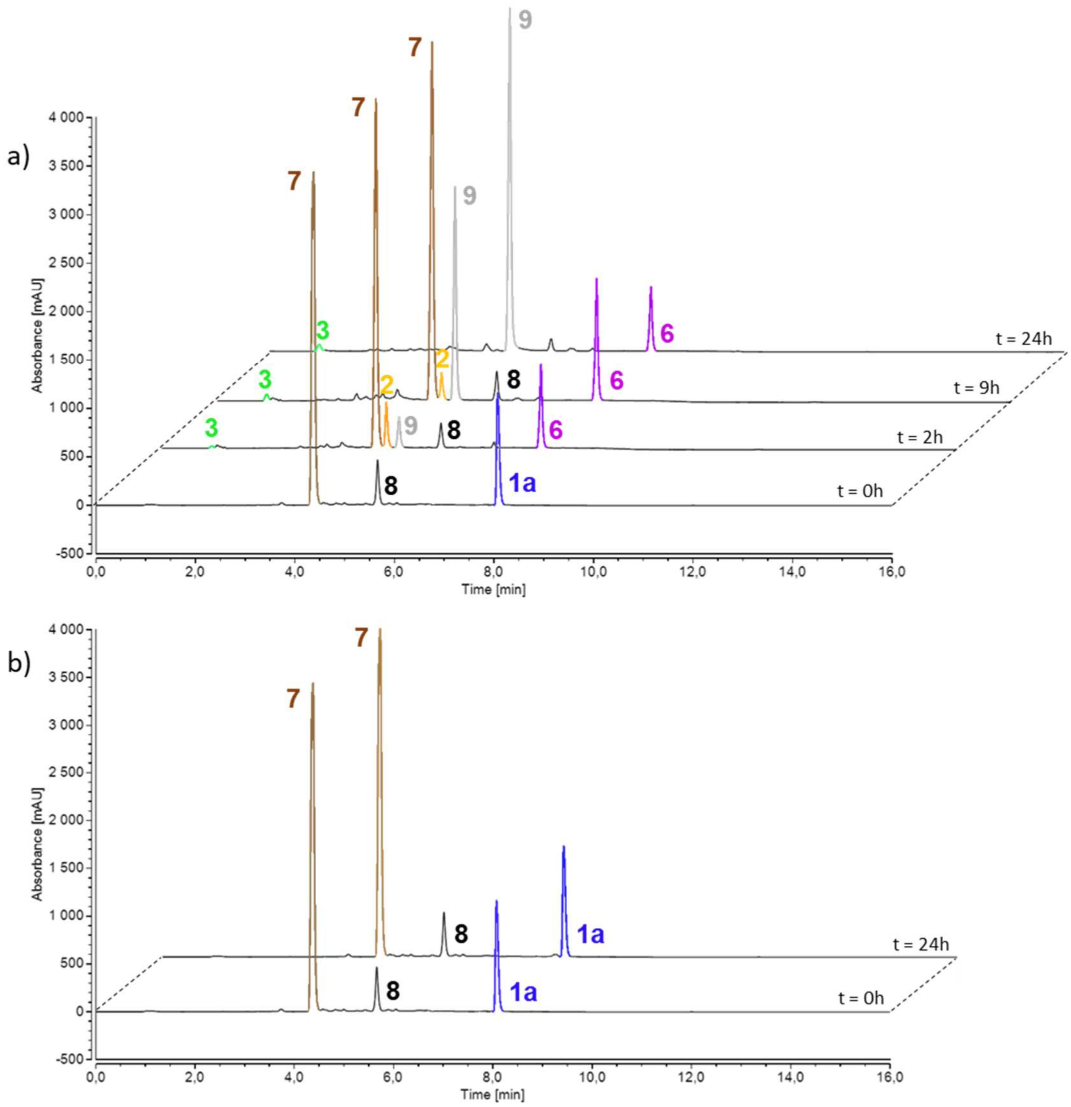

Figure S9: Stacked HPLC showing the reactivity of catenane **1a** a) in the presence of 0.1 equiv. of piperidine; b) without using piperidine. (rt **1a**: 8.1 min, rt **2**: 4.5 min, rt **3**: 1.0 min, rt **6**: 7.6 min, rt **7**: 4.3 min, rt **8**: 5.6 min, rt **9**: 4.7 min).

II.6. Activation of catenane 1b

To a solution of catenane **1b** (2 mg, 1.23 µmol, 1 equiv.) and aniline (0.56 µL, 6.13 µmol, 5 equiv.) in dry EtOH/DMSO 8/2 (110.7 µL) was added a 9.95.10^{-4} M solution of $Pd(PPh_3)_4$ in dry DCE (12.3 µL, 12.3 nmol, 0.01 equiv.). The mixture was heated to 60°C and stirred for 12 hours (step 1 Fig. 5a). Coumarine **7** (2.1 mg, 12.3 µmol, 10 equiv.) and azide **8** (1.8 µL, 14.7 µmol, 12 equiv.) were then added and the reaction was stirred at 60°C (Step 2 fig.5a). The same reaction was realized without using $Pd(PPh_3)_4$. Both reactions were monitored with HPLC.

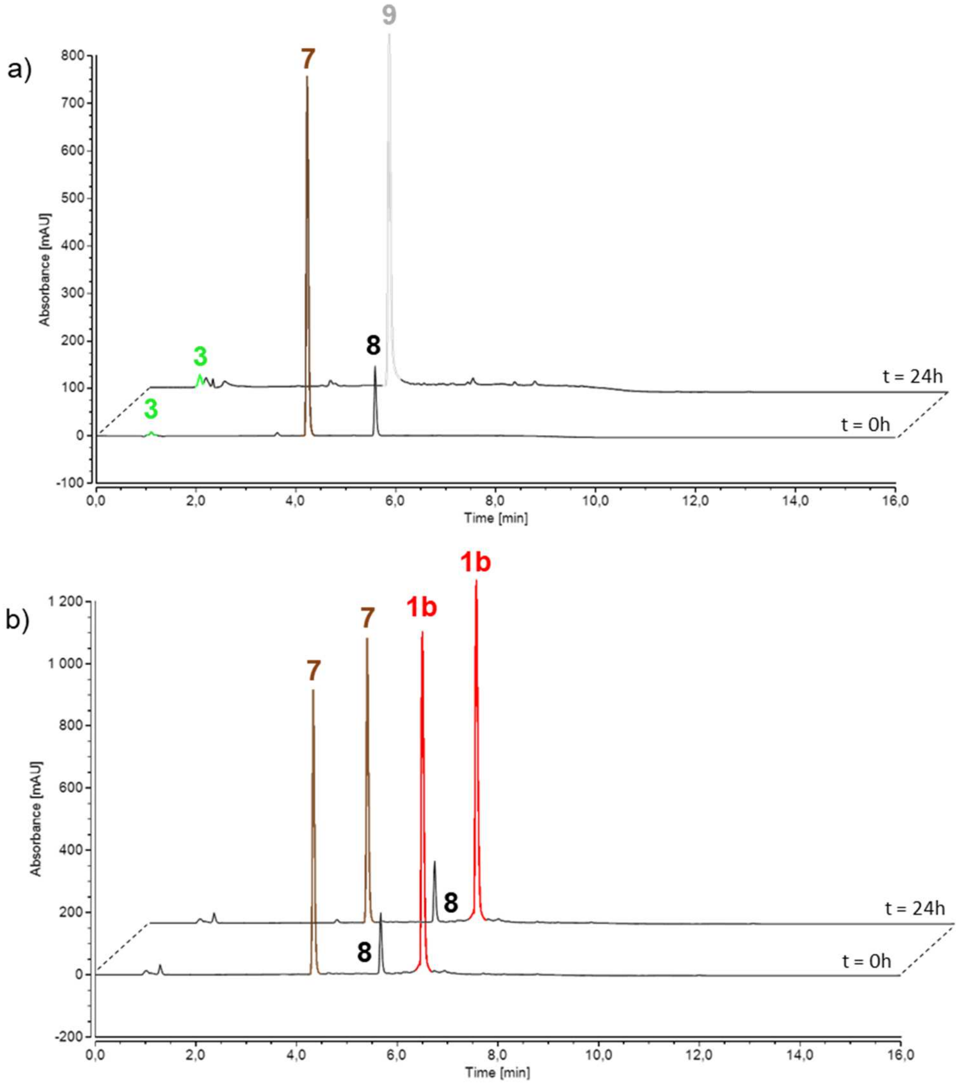

Figure S10: Stacked HPLC showing the reactivity of catenane **1b** after compounds **7** and **8** were added a) after 12 hour with the presence of 0.01 equiv. of $Pd(PPh_3)_4$; b) without using $Pd(PPh_3)_4$. Retention times (min) **1b**: 6.5, **3**: 1.0, **7**: 4.3, **8**: 5.6, **9**: 4.7.

II.7. Catalytic detection of Pd^0

To a solution of catenane **1b** (2 mg, 1.23 µmol, 1 equiv.) and aniline (0.56 µL, 6.13 µmol, 5 equiv.) in dry EtOH/DMSO 8/2 (110.7 µL) was added a decreasing quantity of $Pd(PPh_3)_4$ in dry DCE (12.3 µL) (see table S1). The mixture was heated to 60°C and stirred for 24 hours. Coumarine **7** (2.1 mg, 12.3 µmol, 10 equiv.) and azide **8** (1.8 µL, 14.7 µmol, 12 equiv.) were then added and the reaction was stirred at 60°C and monitored with HPLC.

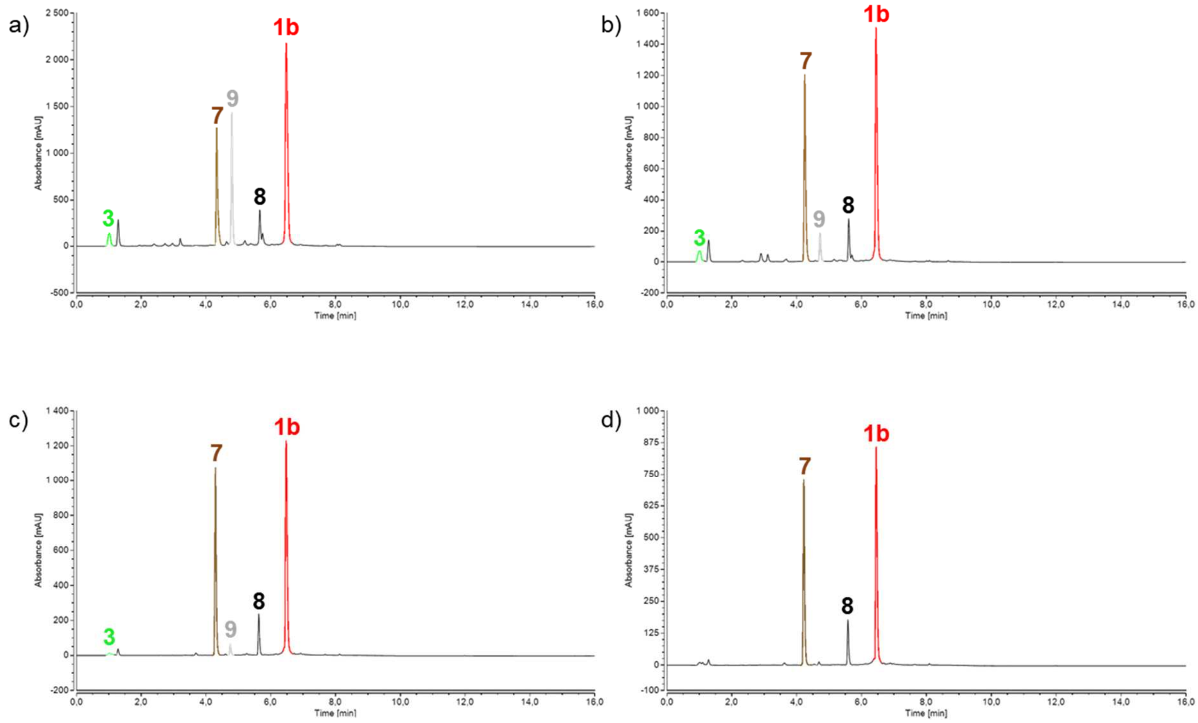

Figure S11: HPLC showing the reactivity of catenane **1b** 24 hours after compounds **7** and **8** were added using a) 1.10^{-3} equiv. of $Pd(PPh_3)_4$; b) 1.10^{-4} equiv. of $Pd(PPh_3)_4$; c) 1.10^{-5} equiv. of $Pd(PPh_3)_4$; d) 1.10^{-6} equiv. of $Pd(PPh_3)_4$. Retention times (min) **1b**: 6.5, **3**: 1.0, **7**: 4.3, **8**: 5.6, **9**: 4.7.

n ($PdPPh_3)_4$)	Equiv. $Pd(PPh_3)_4$	[$Pd(PPh_3)_4$] (mol.L^{-1})	Area of compound **9** (mAU.min)		
1.23 nmol	1.00E-03	1.00E-05	66,533	66.816	65.265
123 pmol	1.00E-04	1.00E-06	8.968	9.287	8.682
12,3 pmol	1.00E-05	1.00E-07	2.736	3.008	2.564
1.23 pmol	1.00E-06	1.00E-08	0.557	0.483	0.396
0	0.00E+00	0.00E+00	0	0	0

Table S1: Formation of compound **9** depending on the quantity of $Pd(PPh_3)_4$ used.

Experimental LOD (SNR 3:1 in HPLC analysis) was found to be 0.286 mAu.min

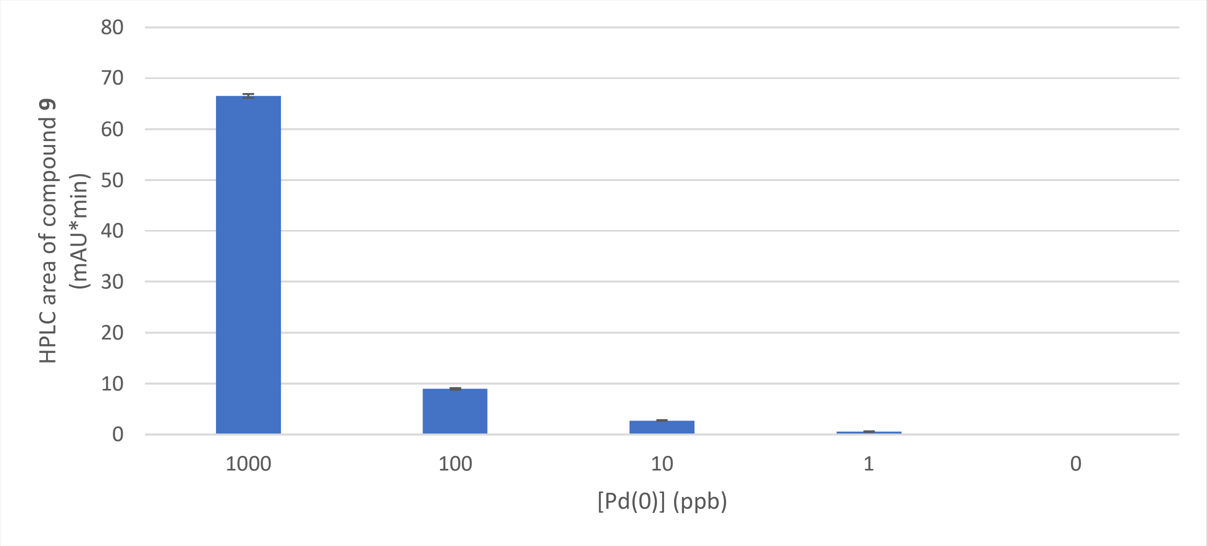

Figure S12: Relative peak area of compound **9** recorded by HPLC/UV as a function of the quantity of Pd(0) introduiced in the reaction medium

II.7. Determination of the dissociation constante (pKd) of catenane 1b

Solutions of **1b** (10^{-7} mol.L^{-1}) in CH_3CN/H_2O (9:1) with an internal standard (L-phenylalanyl-L-phenylalanine $5x10^{-7}$ mol.L^{-1}) were mixed with increasing concentration of KCN in CH_3CN/H_2O (9:1) ranging from 100 to 50 000 equivalents. For each sample UPLC-HRMS analysis allowed to observe the formation of **1c** resulting from the dematallation of **1b.**

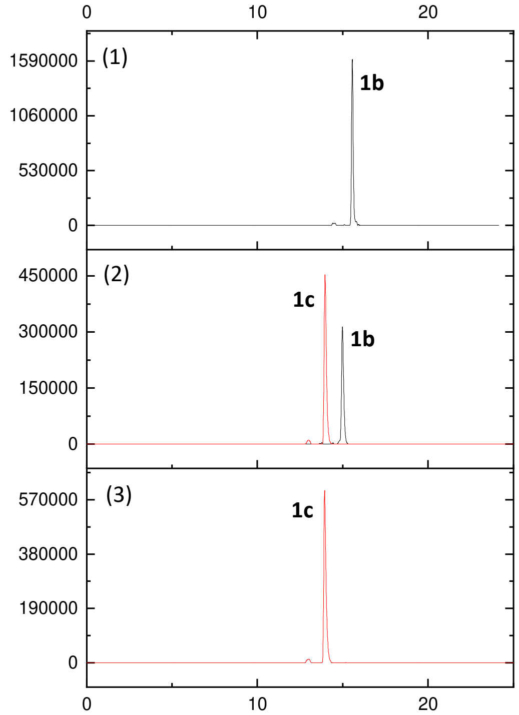

Figure S13: Extracted ion chromatograms for **1b** and **1c** (1) KCN 0 equiv.; (2) KCN 10 000 equiv.; (3) KCN 50 000 equiv.

For each concentration of KCN the areas of **1b** and **1c** observed was correlated with their concentration and a pKd constant was calculated (Kd = [**1c**]^2/[**1b**]).

KCN equiv.	pKd
0	15.55
100	11.62
500	9.01
2 000	7.80
10 000	7.33
50 000	6.73

Table S2: pKd as function of KCN equivalents.

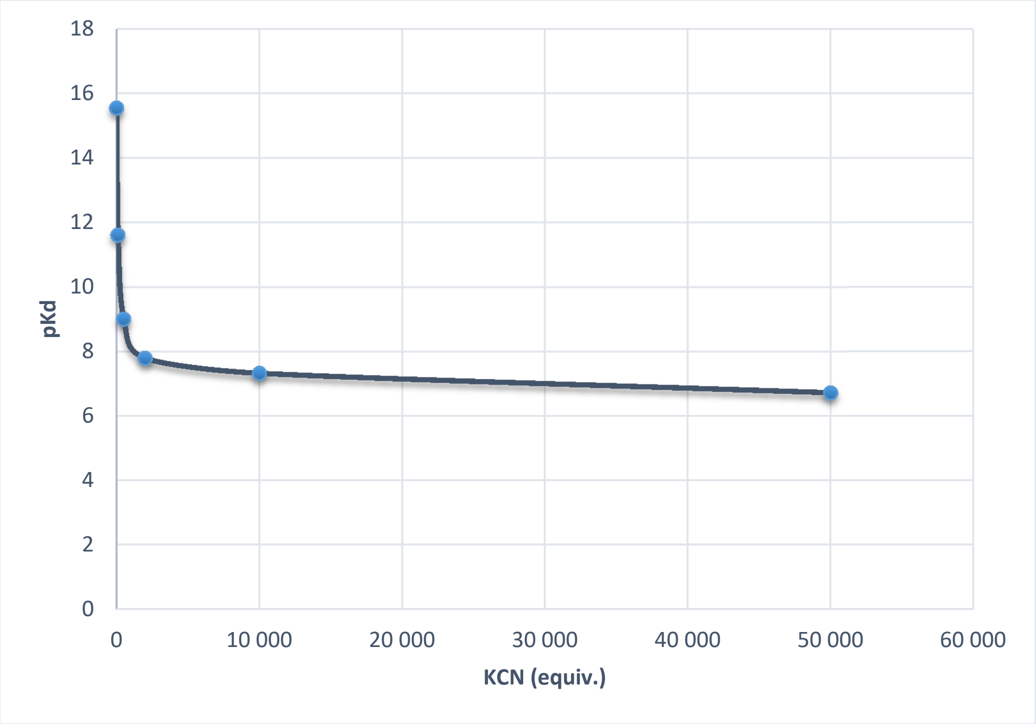

Figure S14: pKd as function of KCN equivalents.

Experimental

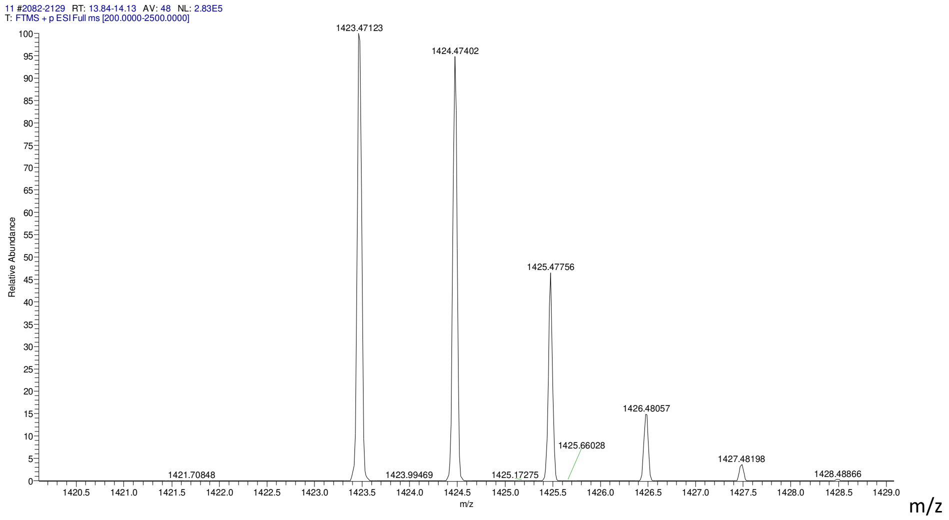

calculated for $C_{80}H_{67}N_{10}O_{16}$ $[M+H]^+$

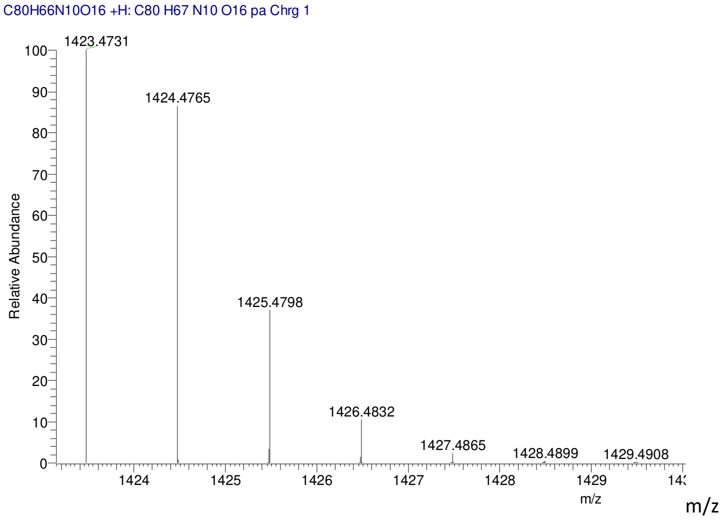

Figure S15: HRMS analysis obtained for the formation of catenane **1c** resulting from the dematallation of **1b.**

III. REFERENCES

1 N. Pairault, A. Bessaguet, R. Barat, L. Frédéric, G. Pieters, J. Crassous, I. Opalinski, S. Papot, *Chem. Sci.,* **2021**, *12*, 2521-2526.

2 V. Bevilacqua, M. King, M. Chaumontet, M. Nothisen, S. Gabillet, D. Buisson, C. Puente, A. Wagner, F. Taran, *Ang. Chem. Int. Ed.*, **2014**, *53*, 5872-5876.

3 R. Erez, D. Shabat, *Org. Biomol. Chem.*, **2008**, *6*, 2669-2672.

4 K. Wang, C.-C. Yee, H. Y. Au-Yeung, *Chem. Sci.*, **2016**, *7*, 2787-.2792.
